# Antibody persistence and immunologic memory in children vaccinated with 4 doses of pneumococcal conjugate vaccines: Results from 2 long-term follow-up studies

**DOI:** 10.1080/21645515.2016.1241919

**Published:** 2016-10-13

**Authors:** Jacek Wysocki, Jerzy Brzostek, Ryszard Konior, Falko G. Panzer, Nancy A. François, Sudheer M. Ravula, Devayani A. Kolhe, Yue Song, Ilse Dieussaert, Lode Schuerman, Dorota Borys

**Affiliations:** a Department of Preventive Medicine, Poznan University School of Medical Sciences, Poznan, Poland; b Pediatrics Department, Zespol Opieki Zdrowotnej w Debicy, Debica, Poland; c Neuro-infection and Pediatric Neurology, John Paul II Hospital, Cracow, Poland; d Gemeinschaftspraxis für Kinder- und Jugendmedizin, Mannheim, Germany; e GSK, Wavre, Belgium; f GSK, Bangalore, India

**Keywords:** antibody persistence, children, immunologic memory, pneumococcal conjugate vaccine, *Streptococcus pneumoniae*

## Abstract

To investigate long-term antibody persistence following the administration of the 10-valent pneumococcal non-typeable *Haemophilus influenzae* protein D conjugate vaccine (PHiD-CV), we present results of 2 follow-up studies assessing antibody persistence following 2 3+1 schedules up to 4 (NCT00624819 – Study A) and 5 years (NCT00891176 – Study B) post-booster vaccination. In Study A, antibody persistence was measured one, 2 and 4 years post-booster in children previously primed and boosted with PHiD-CV, or primed with the 7-valent pneumococcal conjugate vaccine (7vCRM) and boosted with either PHiD-CV or 7vCRM. In Study B, PHiD-CV was co-administered with meningococcal vaccines, and pneumococcal antibody persistence was measured 2, 3 and 5 years post-booster. An age-matched control group, unvaccinated against *Streptococcus pneumoniae*, was enrolled in Study A, allowing assessment of immunologic memory by administration of one dose of PHiD-CV to both primed (4 years post-booster) and unprimed 6-year-old children. Four years post-booster (Study A), antibody concentrations and opsonophagocytic activity (OPA) titers remained higher compared to the pre-booster timepoint, with no major differences between the 3 primed groups. Antibody persistence was also observed in Study B, with minimal differences between groups. The additional PHiD-CV dose administered 4 years post-booster in Study A elicited more robust immune responses in primed children than in unprimed children. Long-term serotype-specific antibody persistence and robust immunologic memory responses observed in these 2 studies suggest induction of long-term protection against pneumococcal disease after PHiD-CV vaccination.

## Introduction

*Streptococcus pneumoniae* is a leading cause of bacterial meningitis, sepsis, pneumonia and acute otitis media.^1^ The greatest disease burden is in children younger than 5 years.^2-4^ Antibodies against capsular polysaccharides of various *S. pneumoniae* serotypes provide serotype-specific protection against pneumococcal infections through antibody-mediated opsonophagocytosis,^5-8^ hence assessment of long term persistence of antibodies is important. However, no correlate of protection has yet been established.

Previous studies showed that infant vaccination with the 10-valent pneumococcal non-typeable *Haemophilus influenzae* protein D conjugate vaccine (PHiD-CV, *Synflorix™*, GSK Vaccines)^9^ led to a decrease in the incidence of invasive pneumococcal disease,^5-7^^,^^10,11^ acute otitis media and community-acquired pneumonia,^12-14^ as well as a marked decrease in antibiotic prescriptions.^15^ PHiD-CV was shown to be immunogenic,^16^ to induce immunologic memory up to 2 years post-booster vaccination,^17,18^ and to have a clinically acceptable safety profile when co-administered with routine pediatric vaccines.^19^ However, antibody persistence after infant vaccination with PHiD-CV beyond 2 years post-booster is unclear.^17,18^ Moreover, as mass vaccination schedules expand, there is a risk that adding further antigens to the schedule could lead to unexpected immune interferences.^20,21^

In order to address these concerns, 2 studies were performed to determine persistence of serotype-specific pneumococcal antibodies and opsonophagocytic activity (OPA) following 2 3+1 vaccination schedules up to 4 and 5 years post-booster vaccination. In the first study (Study A), we assessed antibody persistence at one, 2 and 4 years post-booster in children who received pneumococcal conjugate vaccines (PCVs; PHiD-CV or 7vCRM – the licensed 7-valent PCV, *Prevenar™/Prevnar™*, Pfizer) in the first 2 years of life, and we evaluated immunologic memory 4 years post-booster. In the second study (Study B), we assessed antibody persistence at 2, 3, and 5 years post-booster in children who received PHiD-CV or 7vCRM co-administered with meningococcal serogroup C conjugate vaccines (MenC-CVs).

## Results

**Figure 1.**
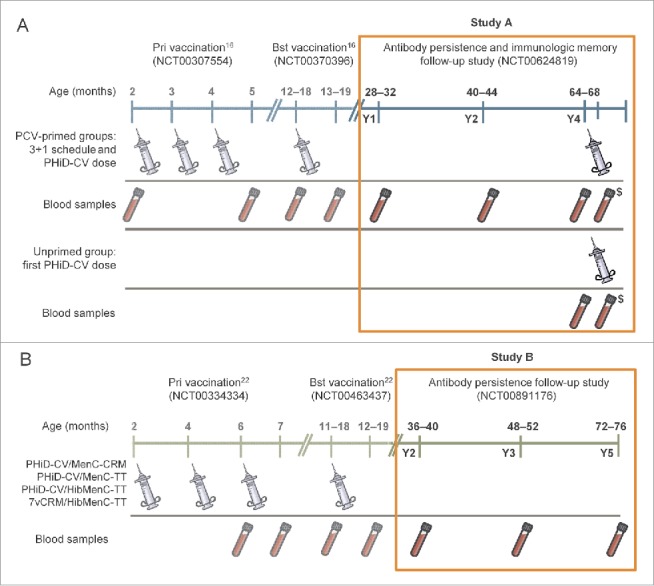
Study procedures for Study A and Study B. Notes: In Study A, all doses of PCVs were co-administered with DTPa-HBV-IPV/Hib at primary and booster vaccination (Finland, Poland and France), except for the 2nd dose in France that was co-administered with DTPa-IPV/Hib. In Study B, the PHiD-CV/MenC-CRM and PHiD-CV/MenC-TT groups received: the meningococcal vaccines as 2-dose primary vaccination in Germany and Spain, 3-dose primary vaccination in Poland (3rd dose received after blood sampling); PHiD-CV was co-administered with DTPa-HBV-IPV/Hib at primary vaccination and DTPa-HBV-IPV/Hib (Germany and Poland) or DTPa-IPV/Hib (Spain) at booster vaccination. In the PHiD-CV/HibMenC-TT and 7vCRM/HibMenC-TT groups, PCVs were co-administered with DTPa-HBV-IPV at primary vaccination and with DTPa-HBV-IPV (Germany and Poland) or DTPa-IPV (Spain) at booster vaccination. ^$^Blood sample collected for immunologic memory assessment 7–10 days after the PHiD-CV dose at Y4, in Study A; Y = number of years following booster vaccination in PCV-vaccinated children; Pri = primary; Bst = booster; PCV = pneumococcal conjugate vaccine.

The design of the 2 studies is displayed with vaccine administration and blood sampling schedules in Fig. 1.

### Demographics

**Figure 2.**
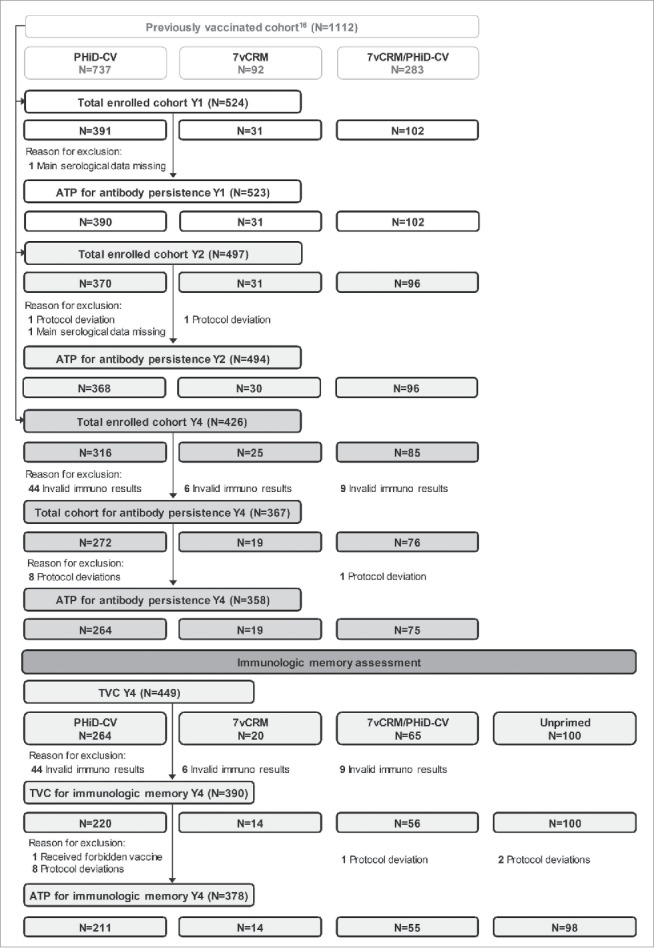
Flow diagram for Study A. N = number of children; Y = number of years following booster vaccination in PCV-vaccinated children; TVC = total vaccinated cohort; ATP = according-to-protocol cohort; PHiD-CV = children previously primed and boosted with PHiD-CV; 7vCRM = children previously primed and boosted with 7vCRM; 7vCRM/PHiD-CV = children previously primed with 7vCRM and boosted with PHiD-CV; Unprimed = age-matched children not previously vaccinated with any pneumococcal vaccine and enrolled as control group for the immunologic memory assessment.

A total of 524 children vaccinated with PCVs in previous studies^16^ were enrolled one year post-booster in Study A, and 426 completed the 4-year follow-up. The according-to-protocol (ATP) cohort for antibody persistence analysis included 523 children one year post-booster, 494 children 2 years post-booster and 358 children 4 years post-booster. At year 4, parents did not provide consent for participation in the immunologic memory assessment for 77 out of 426 children; thus, only 349 primed children were included in the immunologic memory assessment. In addition, at year 4, 100 age-matched children who had not previously received any pneumococcal vaccine (subsequently referred to as “Unprimed”) were enrolled, totaling to 449 children included in the total vaccinated cohort (TVC) for immunologic memory assessment. Out of these, 378 children remained in the ATP cohort for immunologic memory assessment. The number of children in the TVC/ATP cohorts at different timepoints is detailed in Fig. 2, and demographic characteristics of different study groups are presented in Table 1.

**Table 1.** Demographic characteristics of the study population by timepoint (ATP cohorts for the respective timepoints) (Study A and Study B).

Study A	PHiD-CV	7vCRM	7vCRM/PHiD-CV	Unprimed
ATP cohorts for antibody persistence Y1				
N	390	31	102	—
Age (months)/Mean ± SD	29.1 ± 0.87	29.0 ± 0.75	29.1 ± 0.81	—
Sex (female) n (%)	203 (52.1)	14 (45.2)	52 (51.0)	—
ATP cohorts for antibody persistence Y2				
N	368	30	96	—
Age (months)/Mean ± SD	41.1 ± 1.03	41.0 ± 0.83	41.2 ± 1.04	—
Sex (female) n (%)	191 (51.9)	14 (46.7)	50 (52.1)	—
ATP cohorts for antibody persistence Y4				
N	264	19	75	—
Age (months)/Mean ± SD	67.4 ± 0.79	67.3 ± 1.00	67.4 ± 0.84	—
Sex (female) n (%)	140 (53.0)	7 (36.8)	43 (57.3)	—
ATP cohorts for immunologic memory Y4				
N	211	14	55	98
Age (months)/Mean ± SD	67.3 ± 0.83	67.1 ± 1.07	67.2 ± 0.90	65.4 ± 1.30
Sex (female) n (%)	112 (53.1)	5 (35.7)	30 (54.5)	53 (54.1)
Study B	PHiD-CV/MenC-CRM	PHiD-CV/MenC-TT	PHiD-CV/HibMenC-TT	7vCRM/HibMenC-TT
ATP cohorts for antibody persistence Y2				
N	141	146	144	140
Age (months)/Mean ± SD	37.1 ± 1.17	37.2 ± 1.21	37.3 ± 1.18	37.3 ± 1.28
Sex (female) n (%)	71 (50.4)	65 (44.5)	83 (57.6)	71 (50.7)
ATP cohorts for antibody persistence Y3				
N	136	138	133	136
Age (months)/Mean ± SD	48.8 ± 1.25	48.7 ± 1.13	49.2 ± 1.53	48.7 ± 1.22
Sex (female) n (%)	69 (50.7)	60 (43.5)	80 (60.2)	71 (52.2)
ATP cohorts for antibody persistence Y5				
N	128	137	131	134
Age (months)/Mean ± SD	72.8 ± 1.02	72.9 ± 1.09	72.9 ± 1.00	72.9 ± 1.15
Sex (female) n (%)	63 (49.2)	63 (46.0)	77 (58.8)	68 (50.7)

N = number of children; SD = standard deviation; n (%) = number (percentage) of children in a given category; ATP = according-to-protocol; Y = number of years following booster vaccination in PCV-vaccinated children.

**Figure 3.**
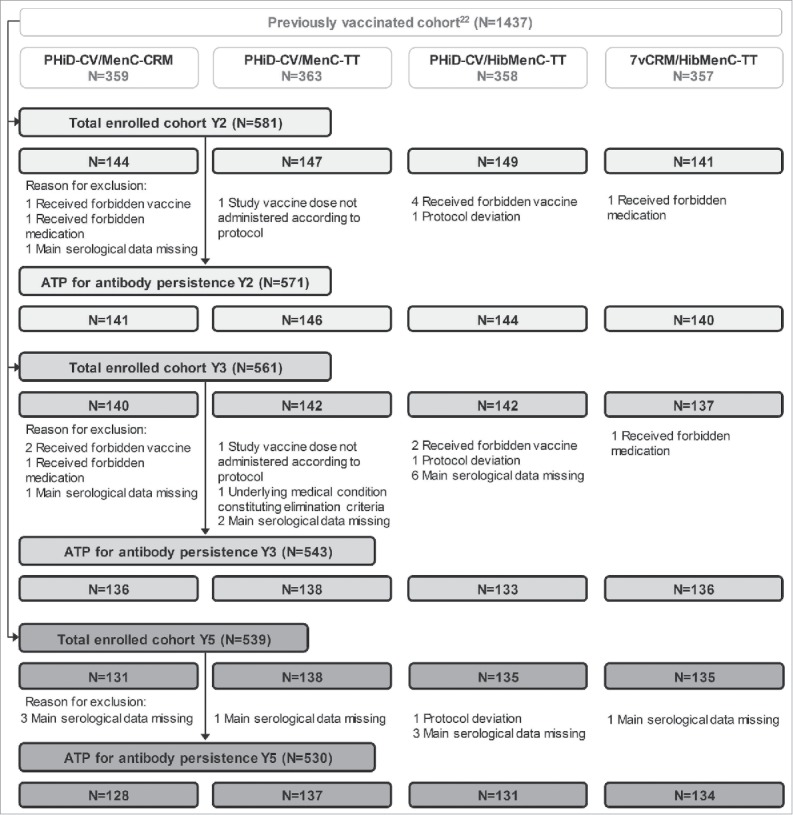
Flow diagram for Study B. N = number of children; Y = number of years following booster vaccination; ATP = according-to-protocol cohort. PHiD-CV/MenC-CRM = children receiving PHiD-CV co-administered with MenC-CRM; PHiD-CV/MenC-TT = children receiving PHiD-CV co-administered with MenC-TT; PHiD-CV/HibMenC-TT = children receiving PHiD-CV co-administered with HibMenC-TT; 7vCRM/HibMenC-TT = children receiving 7vCRM co-administered with HibMenC-TT.

In Study B, a total of 581, 561 and 539 children who had received PHiD-CV or 7vCRM co-administered with MenC-CVs in previous studies^22^ were respectively enrolled 2, 3 and 5 years post-booster, and 539 completed the 5-year follow-up. The ATP cohort for antibody persistence analysis included 571 children 2 years post-booster, 543 children 3 years post-booster and 530 children 5 years post-booster. The number of children enrolled in each cohort at different timepoints is shown in Fig. 3, and demographic characteristics of these cohorts are presented in Table 1.

### Antibody persistence in study A

**Figure 4.**
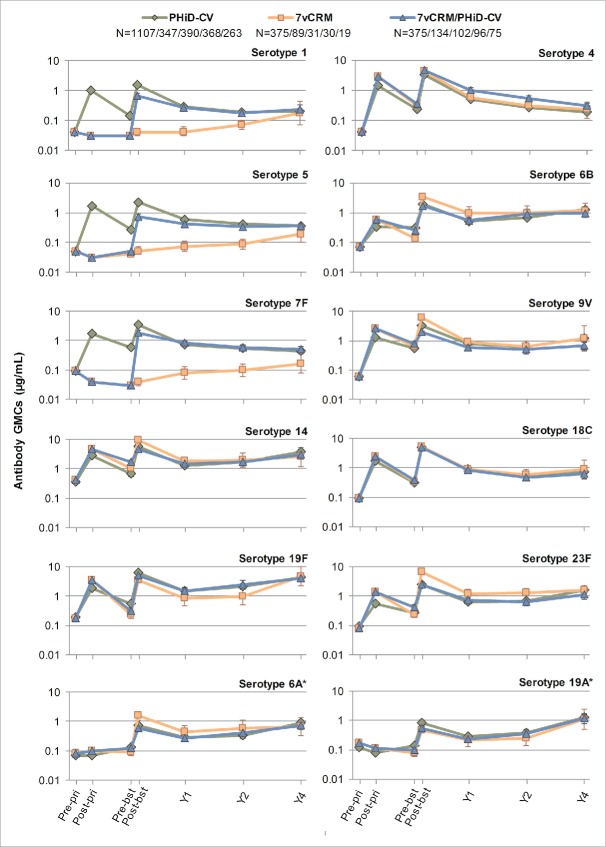
Serotype-specific pneumococcal antibody GMCs following primary and booster vaccination up to year 4 post-booster (Study A) (ATP cohort for immunogenicity and ATP cohorts for antibody persistence at Y1, Y2 and Y4, respectively). *vaccine-related serotypes; GMC = geometric mean concentration; ATP = according-to-protocol; Y = number of years following booster vaccination; N = maximum number of children with available results at primary vaccination/booster vaccination/Y1/Y2/Y4; pre-pri = before the 1st dose of primary vaccination; post-pri = 1 month after the 3rd dose of primary vaccination; pre-bst = before the booster dose; post-bst1 month after the booster dose; error bars indicate 95% confidence intervals.

**Figure 5.**
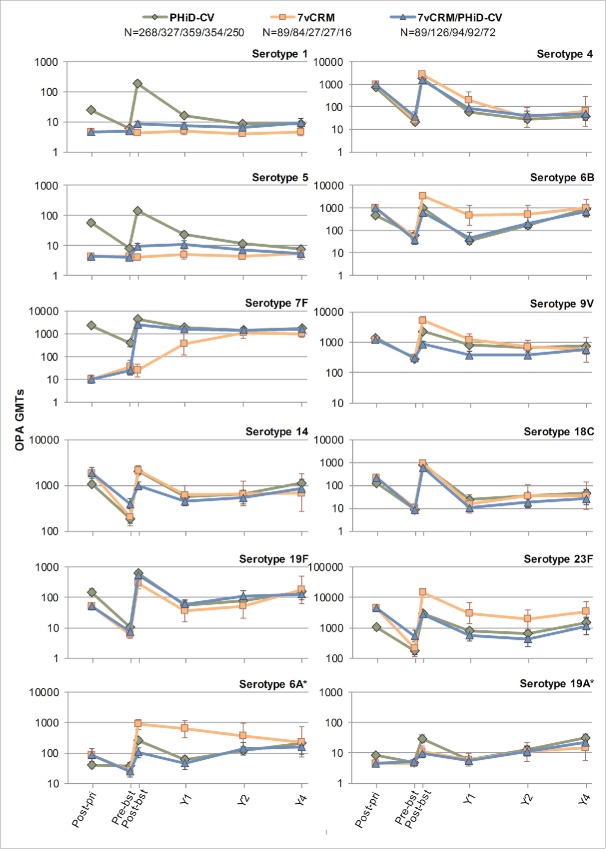
Serotype-specific pneumococcal OPA GMTs post-primary and post-booster vaccination up to year 4 post-booster (Study A) (ATP cohort for immunogenicity and ATP cohorts for antibody persistence at Y1, Y2 and Y4, respectively). *vaccine-related serotypes; OPA = opsonophagocytic activity; GMT = geometric mean titer; ATP = according-to-protocol; Y = number of years following booster vaccination; N = maximum number of children with available results at primary vaccination/booster vaccination/Y1/Y2/Y4; post-pri = 1 month after the 3rd dose of primary vaccination; pre-bst = before the booster dose; post-bst = 1 month after the booster dose; error bars indicate 95% confidence intervals.

In all 3 primed groups (PHiD-CV, 7vCRM and 7vCRM/PHiD-CV), for each of the vaccine serotypes, a decline in antibody geometric mean concentrations (GMCs, Fig. 4) and OPA geometric mean titers (GMTs, Fig. 5) was observed one year post-booster. Two years post-booster, antibody GMCs and OPA GMTs generally remained in the same range as one year post-booster (exceptions were a decrease of antibody GMCs for serotypes 4 and 18C, and an increase for serotypes 14 and 19F). Four years post-booster, antibody GMCs either increased or remained at the same level as year 2, except for serotype 4 for which a decrease was observed (Fig. 4). Few differences could be observed for common serotypes between groups in terms of antibody GMCs and OPA GMTs at all timepoints (Figs. 4 and 5).

Four years post-booster, the percentage of children with antibody concentrations ≥0.20 µg/mL were similar between the PHiD-CV, 7vCRM and 7vCRM/PHiD-CV groups for the 7 common serotypes (except for serotype 4) and serotype 1 (unique to PHiD-CV), regardless of the antibody levels at the previous timepoints. For serotypes 5 and 7F (unique to PHiD-CV), these values appeared to be similar between the PHiD-CV and 7vCRM/PHiD-CV groups (Table S1). At the same timepoint, the percentage of children with antibody concentrations ≥0.05 µg/mL were comparable among groups, except for serotype 7F that appeared to be similar between PHiD-CV receiving groups only (Table S2). The percentages of children with OPA titers ≥8 were within similar ranges between all groups for most serotypes (Table S3).

Although anti-protein D (anti-PD) antibody GMCs were also observed to decline from one month to 4 years post-booster in the PHiD-CV group, their levels remained higher than those in the 7vCRM group at all timepoints (Table 2).

**Table 2.** Seropositivity rates and GMCs for anti-protein D antibodies by timepoint (ATP cohorts for the respective timepoints) (Study A).

	PHiD-CV		7vCRM		7vCRM/PHiD-CV		Unprimed	
	Timepoint	N	% ≥100 EL.U/mL, (LL; UL)	N	% ≥100 EL.U/mL, (LL; UL)	N	% ≥100 EL.U/mL, (LL; UL)	N	% ≥100 EL.U/mL, (LL; UL)
ATP cohorts for immunogenicity at primary/booster vaccination								
Post-pri	1095	99.8 (99.3; 100)	364	17.9 (14.1; 22.2)	364	17.9 (14.1; 22.2)	—	—
Pre-bst	338	94.7 (91.7; 96.8)	73	20.5 (12.0; 31.6)	127	28.3 (20.7; 37.0)	—	—
Post-bst	340	99.4 (97.9; 99.9)	86	18.6 (11.0; 28.4)	134	50.0 (41.2; 58.8)	—	—
ATP cohorts for antibody persistence								
Y1	390	95.6 (93.1; 97.4)	30	36.7 (19.9; 56.1)	102	69.6 (59.7; 78.3)	—	—
Y2	368	92.4 (89.2; 94.9)	29	51.7 (32.5; 70.6)	96	67.7 (57.4; 76.9)	—	—
Y4	261	92.3 (88.4; 95.3)	19	63.2 (38.4; 83.7)	74	73.0 (61.4; 82.6)	—	—
ATP cohorts for immunologic memory Y4								
Pre	208	91.3 (86.7; 94.8)	14	64.3 (35.1; 87.2)	54	66.7 (52.5; 78.9)	95	56.8 (46.3; 67.0)
D7-10-Post	208	99.5 (97.4; 100)	14	100 (76.8; 100)	54	100 (93.4; 100)	96	95.8 (89.7; 98.9)
	N	GMC, EL.U/mL (LL; UL)	N	GMC, EL.U/mL (LL; UL)	N	GMC, EL.U/mL (LL; UL)	N	GMC, EL.U/mL (LL; UL)
ATP cohorts for immunogenicity at primary/booster vaccination								
Post-pri	1095	1529.9 (1452.3; 1611.8)	364	66.1 (61.6; 70.9)	364	66.1 (61.6; 70.9)	—	—
Pre-bst	338	556.4 (494.7; 625.7)	73	72.3 (59.3; 88.0)	127	78.1 (67.8; 89.9)	—	—
Post-bst	340	2887.6 (2573.7; 3239.8)	86	75.3 (60.0; 94.4)	134	125.5 (103.4; 152.4)	—	—
ATP cohorts for antibody persistence								
Y1	390	822.1 (731.5; 923.9)	30	93.9 (66.6; 132.3)	102	193.6 (155.9; 240.4)	—	—
Y2	368	573.2 (509.6; 644.8)	29	116.7 (80.9; 168.3)	96	157.5 (128.7; 192.8)	—	—
Y4	261	372.4 (329.6; 420.9)	19	144.9 (86.3; 243.2)	74	161.4 (128.4; 203.0)	—	—
ATP cohorts for immunologic memory Y4								
Pre	208	374.3 (324.8; 431.3)	14	133.3 (78.3; 227.0)	54	141.6 (109.1; 183.8)	95	106.0 (91.1; 123.4)
D7-10-Post	208	2106.0 (1806.7; 2454.9)	14	718.2 (442.5; 1165.7)	54	680.7 (522.9; 886.2)	96	382.9 (320.7; 457.2)

GMC = geometric mean antibody concentration; ATP = according-to-protocol; EL.U = ELISA units; % ≥100 EL.U/mL = percentage of children with antibody concentration ≥ 100 EL.U/mL; N = number of children with available results; LL = lower limit of the 95% confidence interval; UL = upper limit of the 95% confidence interval; post-pri = 1 month after the 3rd dose of primary vaccination; pre-bst = before the booster dose; post-bst = 1 month after the booster dose; Y = number of years following booster vaccination in PCV-vaccinated children; pre = before the additional PHiD-CV dose in PCV-vaccinated children or before the first PHiD-CV dose in the Unprimed group; D7-10-Post = 7–10 days after the additional dose in PCV-vaccinated children or 7–10 days after the first dose in the Unprimed group.

### Antibody persistence in study B

**Figure 6.**
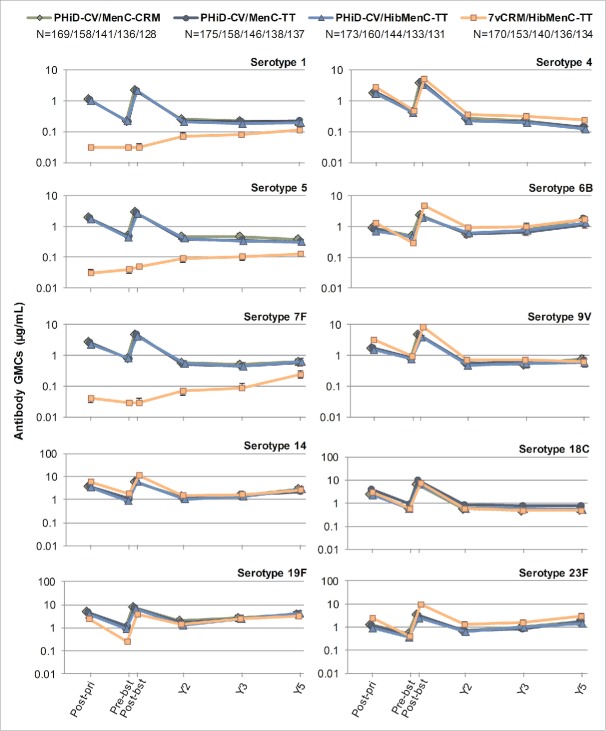
Serotype-specific pneumococcal antibody GMCs post-primary and post-booster vaccination up to year 5 post-booster (Study B) (ATP cohort for immunogenicity and ATP cohorts for antibody persistence at Y2, Y3 and Y5, respectively). GMC = geometric mean concentration; ATP = according-to-protocol; Y = number of years following booster vaccination; N = maximum number of children with available results at primary vaccination/booster vaccination/Y2/Y3/Y5; post-pri = 1 month after the 3rd dose of primary vaccination; pre-bst = before the booster dose; post-bst = 1 month after the booster dose; error bars indicate 95% confidence intervals.

**Figure 7.**
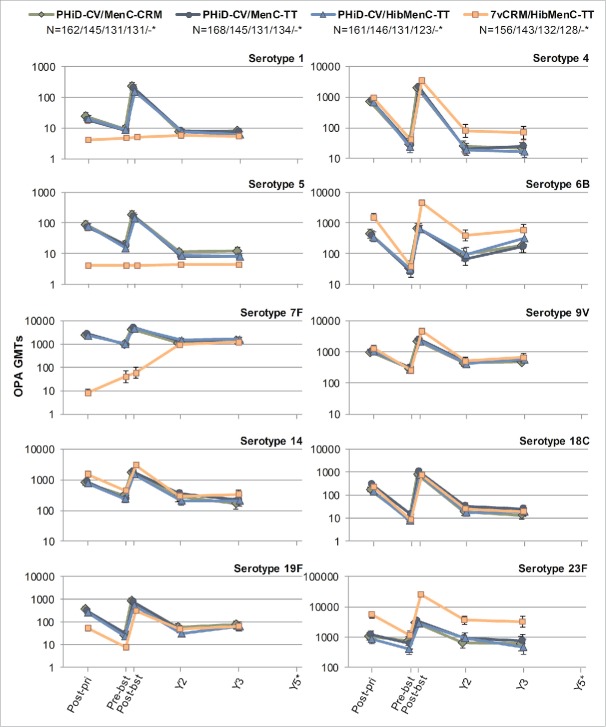
Serotype-specific pneumococcal OPA GMTs post-primary and post-booster vaccination up to year 3 post-booster (Study B) (ATP cohort for immunogenicity and ATP cohorts for antibody persistence at Y2 and Y3, respectively). *testing of *Streptococcus pneumoniae* opsonophagocytic activity (OPA) was not performed at year 5; GMT = geometric mean titer; ATP = according-to-protocol; Y = number of years following booster vaccination; N = maximum number of children with available results at primary vaccination/booster vaccination/Y2/Y3; post-pri = 1 month after the 3rd dose of primary vaccination; pre-bst = before the booster dose; post-bst = 1 month after the booster dose; error bars indicate 95% confidence intervals.

For all vaccine serotypes, antibody GMCs declined from one month to 2 years post-booster (Fig. 6), then stabilized up to 5 years post-booster (except for serotype 4 where antibody GMCs continued to decrease in all groups). Similarly, OPA GMTs declined from one month to 2 years post-booster (Fig. 7), then stabilized up to 3 years post-booster, the last timepoint at which OPA GMTs were evaluated. When comparing the 4 groups, no major differences were observed in terms of antibody GMCs and OPA GMTs for common serotypes at all timepoints, except for consistent trend of higher OPA GMT for serotypes 4, 6B and 23F in 7vCRM/HibMenC-TT group post-booster vaccination (Figs. 6 and 7).

In PHiD-CV vaccinees, the percentages of children with antibody concentrations or OPA titers above the predefined thresholds decreased at each subsequent timepoint after the booster dose for most vaccine serotypes, but values remained similar between groups, except for serotype 5 (percentages of children with OPA titers ≥8 ) and serotype 18C (percentages of children with antibody concentration and OPA titers above thresholds) (Tables S4 and S5).

Data on antibody persistence from Study A and Study B did not reveal major differences, suggesting that co-administration of MenC-CVs did not alter persistence of immune responses to PHiD-CV.

Antibody GMCs against PD tended to decline with time from the post-booster timepoint in all 3 groups receiving PHiD-CV, but remained consistently higher than that in the 7vCRM group up to 5 years post-booster (Table 3).

**Table 3.** Seropositivity rates and GMCs for anti-protein D antibodies by timepoint (ATP cohorts for the respective timepoints) (Study B).

	PHiD-CV/MenC-CRM		PHiD-CV/MenC-TT		PHiD-CV/HibMenC-TT		7vCRM/HibMenC-TT	
	Timepoint	N	% ≥100 EL.U/mL, (LL; UL)	N	% ≥100 EL.U/mL, (LL; UL)	N	% ≥100 EL.U/mL, (LL; UL)	N	% ≥100 EL.U/mL, (LL; UL)
ATP cohorts for immunogenicity at primary/booster vaccination								
Post-pri	168	100 (97.8; 100)	174	100 (97.9; 100)	173	99.4 (96.8; 100)	163	23.3 (17.1; 30.6)
Pre-bst	147	98.0 (94.2; 99.6)	153	96.1 (91.7; 98.5)	147	93.9 (88.7; 97.2)	146	40.4 (32.4; 48.8)
Post-bst	158	100 (97.7; 100)	152	99.3 (96.4; 100)	160	100 (97.7; 100)	148	44.6 (36.4; 53.0)
ATP cohorts for antibody persistence								
Y2	140	94.3 (89.1; 97.5)	145	94.5 (89.4; 97.6)	144	94.4 (89.3; 97.6)	139	49.6 (41.1; 58.2)
Y3	136	93.4 (87.8; 96.9)	138	92.8 (87.1; 96.5)	129	89.1 (82.5; 93.9)	132	56.8 (47.9; 65.4)
Y5	128	88.3 (81.4; 93.3)	137	86.9 (80.0; 92.0)	131	86.3 (79.2; 91.6)	133	55.6 (46.8; 64.2)
	N	GMC, EL.U/mL (LL; UL)	N	GMC, EL.U/mL (LL; UL)	N	GMC, EL.U/mL (LL; UL)	N	GMC, EL.U/mL (LL; UL)
ATP cohorts for immunogenicity at primary/booster vaccination								
Post-pri	168	2114.0 (1847.6; 2418.8)	174	1715.5 (1494.9; 1968.7)	173	1726.7 (1493.3; 1996.7)	163	72.3 (64.5; 81.1)
Pre-bst	147	779.2 (665.2; 912.8)	153	666.6 (563.2; 789.1)	147	635.8 (529.9; 763.0)	146	96.0 (82.9; 111.1)
Post-bst	158	3106.0 (2693.8; 3581.3)	152	2598.4 (2206.9; 3059.4)	160	2679.3 (2305.5; 3113.6)	148	96.4 (84.1; 110.5)
ATP cohorts for antibody persistence								
Y2	140	494.6 (414.0; 591.0)	145	444.8 (373.2; 530.1)	144	439.8 (366.4; 527.9)	139	100.3 (87.6; 114.9)
Y3	136	401.1 (338.3; 475.6)	138	387.2 (326.7; 458.9)	129	334.8 (277.7; 403.7)	132	107.9 (94.6; 123.1)
Y5	128	284.7 (239.5; 338.4)	137	278.0 (236.3; 327.1)	131	266.1 (222.2; 318.8)	133	105.2 (92.4; 119.8)

GMC = geometric mean antibody concentration; ATP = according-to-protocol; EL.U = ELISA units; % ≥100 EL.U/mL = percentage of children with antibody concentration ≥ 100 EL.U/mL; N = number of children with available results; LL = lower limit of the 95% confidence interval; UL = upper limit of the 95% confidence interval; post-pri = 1 month after the 3rd dose of primary vaccination; pre-bst = before the booster dose; post-bst = 1 month after the booster dose; Y = number of years following booster vaccination.

### Immunologic memory assessment by measuring the early immune responses 7–10 days following an additional dose of PHiD-CV 4 years post-booster in study A

**Figure 8.**
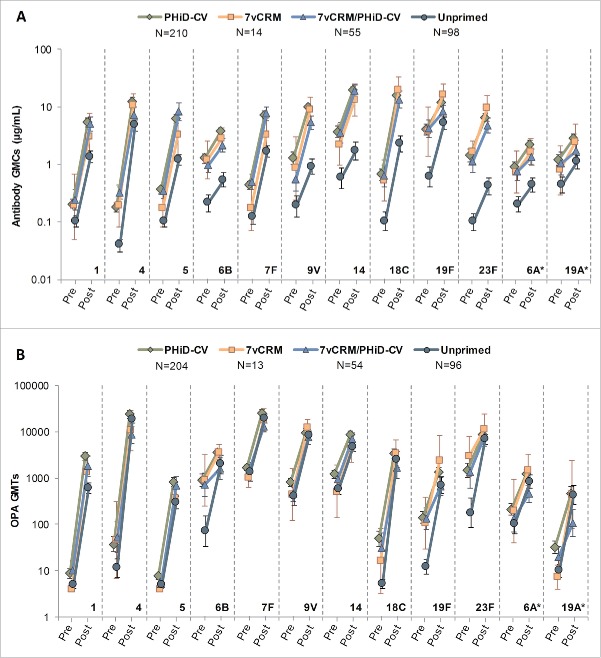
Serotype-specific pneumococcal antibody GMCs (A) and OPA GMTs (B) before and 7–10 days after the additional PHiD-CV dose in PCV-vaccinated children or the first PHiD-CV dose in the Unprimed group (Study A) (ATP cohort for immunologic memory at year 4). *vaccine-related serotypes; PCV = pneumococcal conjugate vaccine; ATP = according-to-protocol; GMC = geometric mean concentration; OPA = opsonophagocytic activity; GMT = geometric mean titer; N = maximum number of children with available results; pre = before the additional PHiD-CV dose in PCV-vaccinated children or before the first PHiD-CV dose in the Unprimed group; post = 7–10 days after the additional PHiD-CV dose in PCV-vaccinated children or 7–10 days after the first PHiD-CV dose in the Unprimed group; error bars indicate 95% confidence intervals.

For each vaccine and vaccine-related (6A and 19A) serotype, substantial increases in the antibody GMCs and OPA GMTs were observed in all groups 7–10 days post-additional dose, compared to pre-vaccination (Fig. 8).

For most serotypes, the additional dose elicited more robust immune responses (antibody GMCs and OPA GMTs) to the 10 vaccine pneumococcal serotypes at 7–10 days post-vaccination in the 3 primed groups compared to those observed in the Unprimed group. This is indicative of an anamnestic immune response in primed children (Fig. 8). For serotypes 1, 5 and 7F, antibody GMCs at 7–10 days post-vaccination were similar in the PHiD-CV and 7vCRM/PHiD-CV groups (Fig. 8A).

Anti-PD antibody GMCs were higher at 7–10 days post-vaccination in the PHiD-CV group than in the 3 other groups, where similar anti-PD antibody GMCs were observed (Table 2).

### Safety

In Study A, one serious adverse event (SAE, bronchopneumonia) was reported in one child during the first year of follow-up. It resolved without sequelae and it was considered by the investigator not causally related to the vaccination. In all groups, the additional dose of PHiD-CV administered in 6-year-olds was well tolerated and no SAEs were reported.

No SAEs that were considered to be causally related to the vaccination or study participation were reported in Study B.

## Discussion

Vaccine-elicited pneumococcal antibodies have been shown to persist up to 4 years^23^ and an anamnestic immune response has been seen 5 years^24^ after administration of the 7vCRM vaccine. Here, we observed antibody persistence for all vaccine pneumococcal and vaccine-related serotypes (6A and 19A) 4 (Study A) and 5 years (Study B) after the PHiD-CV booster dose; previous studies assessed antibody persistence up to 2 years post-booster.^17,18^

As expected, post-booster antibody GMCs and OPA GMTs tended to decrease gradually over time. However, children with declining post-vaccination antibody concentrations and OPA titers do not necessarily become susceptible to pneumococcal disease. In a previous study, an antibody decline was noted after vaccination with 9vCRM, but vaccine efficacy against invasive pneumococcal disease was still 77.8% 6 years post-vaccination.^25^ The induction of immunologic memory following vaccination is considered to be a key factor in the long-term protection against invasive pneumococcal disease.

For each of the common vaccine serotypes, antibody GMCs, OPA GMTs and percentages of children with antibody concentrations and OPA titers above the predefined thresholds tended to be similar between primed groups up to 4 (Study A) or 5 years (Study B) post-booster, regardless of antibody levels in the previous years. Antibody persistence was observed against all serotypes analyzed, including those not commonly found in the nasopharynx and for which the limited natural exposure is thus unlikely to have contributed to the persistence of antibodies.^26^

The primary mechanism of defense against *S. pneumoniae* is antibody-mediated opsonophagocytosis.^27^ Our results show that functional antibodies persist up to 4 years after booster vaccination with PHiD-CV. Limited data on OPA persistence is available in the literature, and we have not found any published reports of persistence of OPA responses on such a long time-scale. Therefore, our findings that there were limited differences between groups in both studies and that the co-administration of MenC-CVs did not affect long-term OPA persistence are of interest.

One of the key features of conjugate vaccines is that they facilitate the induction of immunologic memory and therefore, through the rapid induction of a robust anamnestic response upon subsequent pathogen exposure, contribute to long-term protection against bacterial infections. The induction of immune memory following previous primary and/or booster vaccination with PHiD-CV was assessed in Study A through the measurement of early immune responses following the administration of an additional dose of PHiD-CV 4 years post-booster. Higher antibody GMCs and OPA GMTs in the PHiD-CV primed/boosted children compared to the unprimed children provide evidence of immunologic memory induced by PHiD-CV.

Persistence of anti-PD antibodies has also been observed in both studies. Our findings indicate that a single previous dose of PHiD-CV is not sufficient to prime the immune response, as anti-PD antibody GMCs following administration of an additional PHiD-CV dose 4 years post-booster were essentially the same in the 7vCRM/PHiD-CV and 7vCRM groups as well as in the Unprimed group. Even though the clinical significance of antibody concentrations against PD has not been established, a previous study has shown that the efficacy of an 11-valent pneumococcal PD-conjugate vaccine against episodes of acute otitis media caused by non-typeable *H. influenzae* was 35.3% (95% CI, 1.8–57.4).^28^

The studies presented here have several limitations. Comparison of the results between groups in Study A should be done with caution because study objectives were exploratory (no pre-defined criteria and no correction for multiplicity). Also, the small sample size of the 7vCRM group was a potential limitation. A limitation of Study B was the lack of assessment of OPA responses 5 years after the booster dose.

In conclusion, persistence of immune responses induced by PHiD-CV was observed 4 to 5 years post-booster in 6- and 7-year-old children. Moreover, an additional dose administered in 6-year-olds elicited a more robust immune response for all vaccine serotypes and vaccine-related serotypes (6A and 19A), in primed children than in age-matched unprimed children, indicative of an anamnestic response in primed children. These results suggest that PHiD-CV protective efficacy may extend until at least 4 years after booster vaccination.

## Materials and methods

### Study design, vaccines and participants

Study A was a Phase III, open-label, controlled multicenter long-term follow-up study with 4 groups (Fig. 1) performed in 6 centers in Poland between March 2008 and November 2011 (ClinicalTrials.gov; NCT00624819). This study was a continuation of a primary vaccination study (NCT00307554) and a booster study (NCT00370396) performed in Finland, France and Poland.^16^ The study population at enrollment (one year after the booster dose) was composed of healthy children aged 28–32 months who were previously vaccinated according to a 3 + 1 schedule at 2, 3, 4 and 12–18 months of age with either PHiD-CV (PHiD-CV group), 7vCRM (7vCRM group), or 7vCRM primary + PHiD-CV booster (7vCRM/PHiD-CV group), co-administered with diphtheria-tetanus-acellular pertussis-hepatitis B-inactivated poliomyelitis and *H. influenzae* type b vaccine ([DTPa-HBV-IPV/Hib], *Infanrix hexa™*, GSK Vaccines).^16^ For all children, informed consent was obtained at study entry at year one post-booster and an additional Informed Consent Form was acquired for the children involved in the immunologic memory assessment. Anti-pneumococcal IgG antibodies and OPA were measured at 1, 2, and 4 years post-booster dose for antibody persistence assessment. For assessment of immunologic memory, an additional dose of PHiD-CV was offered at year 4 post-booster to each primed child who entered this study phase, and a catch-up dose of PHiD-CV to enrolled age-matched 64–68 month-old healthy children who had not previously received any pneumococcal vaccines (Unprimed group).

The study vaccine PHiD-CV (0.5 mL per dose) contained 1 µg of each capsular polysaccharide from pneumococcal serotypes 1, 5, 6B, 7F, 9V, 14 and 23F, and 3 µg of serotype 4 conjugated to non-typeable *H. influenzae* PD, 3 µg of capsular polysaccharide of serotype 18C conjugated to tetanus toxoid, and 3 µg of capsular polysaccharide of serotype 19F conjugated to diphtheria toxoid. The vaccine (lot# ASPNA060F) was administered in the deltoid muscle of the participants in Study A.

Study B was a Phase III, open label, controlled multicenter long-term follow-up study with 4 parallel groups (Fig. 1) performed in 24 centers in Germany, Poland and Spain between May 2009 and November 2012 (NCT00891176) as a continuation of a primary vaccination study (NCT00334334) and a booster study (NCT00463437).^19,22,^^29^ Details of the vaccination schedule, vaccine composition and study groups can be found in Fig. 1, and have been described previously.^19,22,^^29^ Briefly, children received 3+1 doses of PHiD-CV or 7vCRM co-administered with MenC-CVs at 2, 4, 6 and 11–18 months of age. PHiD-CV was co-administered with either MenC-CRM_197_ (*Meningitec*™, Nuron Biotech), MenC-TT (*NeisVac-C*™, Pfizer) or HibMenC-TT (*Menitorix*™, GSK Vaccines), while 7vCRM was only co-administered with HibMenC-TT. Anti-pneumococcal IgG antibodies were measured at 2, 3 and 5 years post-booster dose and OPA responses were determined 2 and 3 years post-booster dose for antibody persistence assessment. No study vaccine was administered during this long-term follow-up.

### Immunogenicity assessment

For the assessment of antibody persistence, blood samples were taken at the previously described timepoints for both studies. For the assessment of immunologic memory in Study A, blood samples were taken prior to and 7–10 days after the PHiD-CV dose administered to primed and unprimed 6-year-old children. After blood centrifugation and serum separation, samples were stored at or below −20 °C until analysis.

IgG antibodies against 10 pneumococcal vaccine serotypes (1, 4, 5, 6B, 7F, 9V, 14, 18C, 19F, 23F) and 2 vaccine-related serotypes (6A and 19A) were measured by 22F-inhibition enzyme-linked immunosorbent assay (ELISA; in-house assay at GSK Biologicals laboratory or validated laboratory designated by GSK Biologicals), as described previously.^30,31^ The percentage of children with antibody concentrations above 0.05 µg/mL and 0.20 µg/mL are presented here. A concentration of 0.20 µg/mL measured using GSK's 22F-inhibition ELISA assay corresponds to a concentration of 0.35 µg/mL as determined using the World Health Organization reference ELISA without 22F adsorption,^30^ which is agreed to represent the non-inferiority threshold for licensure of new PCVs for active immunization against invasive pneumococcal disease.

OPA was measured by a killing assay using an HL-60 cell line. The results were presented as the dilution of serum (opsonic titer) able to sustain 50% killing of live pneumococci under assay conditions (in-house assay at a GSK Biologicals laboratory or validated laboratory designated by GSK Biologicals). The cut-off of the assay was an opsonic titer of 8 as previously described.^32^

IgG antibodies to the *H. influenzae* PD were measured by an ELISA assay developed by GSK Biologicals, with the nonlipidated PD as coating material. Concentration of specific PD antibodies was determined using a standard reference serum. The cut-off of the assay was 100 ELISA units (EL.U) per milliliter.

### Safety reporting

Vaccine-related SAEs were to be recorded from the end of the booster vaccination study up to the end of the follow-up studies for Studies A and B.

In addition, in Study A, following administration of PHiD-CV, parents or guardians were asked to complete diary cards to report local (pain, redness and swelling) and general symptoms (fever, irritability/fussiness, drowsiness and loss of appetite) over the first 4 days after PHiD-CV vaccination, and unsolicited adverse events (AEs) over 31 days after vaccination. Safety data for the unprimed children have been reported previously.^33^ All SAEs following the PHiD-CV vaccination were to be recorded up to the study end.

### Statistical analysis

The statistical analyses were performed using the Statistical Analysis System (SAS) Drug and Development (SDD) Web portal version 3.5 and SAS version 9.22.

Immunogenicity analyses were performed based on the ATP cohorts for antibody persistence year one, year 2 and year 4 and on the ATP cohort for immunologic memory year 4 in Study A and on the ATP cohorts for antibody persistence year 2, year 3 and year 5 in Study B, which included only children who complied with study procedures and had results available for at least one vaccine antigen.

Antibody GMCs, OPA GMTs and percentages of children reaching predefined immunologic thresholds or cut-offs were calculated with 95% confidence intervals (CIs) for each vaccine pneumococcal serotype and for vaccine-related serotypes 6A and 19A. Anti-PD antibody GMCs and seropositivity rates were also calculated with 95% CIs.

Antibody concentrations and OPA titers below the assay cut-off were given an arbitrary value of half of the cut-off for the purpose of GMC and GMT calculations. The safety analysis was performed based on the TVC.

### Ethics

The study protocol was approved by ethics review committees of participating centers. Written informed consent was obtained from the parents or legal guardians before enrollment. Overall, the studies were undertaken in accordance with principles of Good Clinical Practice (GCP) guidelines and the Declaration of Helsinki. During the course of the study, whenever potential or actual issues with regard to the conduct of the study were identified, either via site monitoring activities or brought to GSK Biologicals’ attention by other oversight mechanisms, these issues were investigated and appropriate corrective and/or preventive actions were taken.

In Study A, obvious incoherence in the immunogenicity results per consecutive timepoints of the same children was detected in 3 out of 6 study sites. Based on evidence of a systematic error of mislabelling in 2 sites, individual serology results in the database between the 2 timepoints were switched (a total of 110 children). For the third clinical site, no evidence of systematic error was found to explain the incoherence, so the data (obtained from 59 children) from this site were excluded from any immunogenicity analysis.

In Study B, some deviations in terms of study documentation and adherence to the study protocol were identified. At one of the study centers some additional GCP deviations were noted such as the finding that annual reports and updates of informed consent had not been sent to regulatory authorities, as required by local law. However, after a full investigation by a GSK department independent of the study team and discussion with local regulatory agencies, it was determined that these findings did not have an impact on the safety of children or on data integrity.

## Trademarks

Synflorix, Infanrix hexa and Menitorix are trademarks of the GSK group of companies. Prevenar/Prevnar and NeisVac-C are trademarks of Pfizer. Meningitec is a trademark of Nuron Biotech.

## Supplementary Material

KHVI_A_1241919_Supplementary_material.zip

## Abbreviations

7vCRM: 7-valent pneumococcal conjugate vaccine
AE: adverse event
ATP: according-to-protocol
CI: confidence interval
DTPa-HBV-IPV/Hib: diphtheria-tetanus-acellular pertussis-hepatitis B-inactivated poliomyelitis and *Haemophilus influenzae* type b vaccine
ELISA: enzyme-linked immunosorbent assay
EL.U: ELISA units
GCP: Good Clinical Practice
GMC: geometric mean concentration
GMT: geometric mean titer
MenC-CV: meningococcal serogroup C conjugate vaccine
OPA: opsonophagocytic activity
PCV: pneumococcal conjugate vaccine
PD: protein D
PHiD-CV: pneumococcal non-typeable *Haemophilus influenzae* protein D conjugate vaccine
SAE: serious adverse event
TVC: total vaccinated cohort
