# Supplementary material for: Antibody persistence and immunologic memory in children vaccinated with 4 doses of pneumococcal conjugate vaccines: Results from 2 long-term follow-up studies
Source: Hum Vaccin Immunother. 2016 Oct 13;13(3):661–75. doi: 10.1080/21645515.2016.1241919 (PMC5360132; doi:10.1080/21645515.2016.1241919)
Supplement: KHVI_A_1241919_Supplementary_material.zip [file khvi-13-03-1241919-s001.zip › 2016HV0225R-s02.docx]

**Antibody persistence and immunologic memory in children vaccinated with four doses of pneumococcal conjugate vaccines: results from two long-term follow-up studies**

Jacek Wysocki^1^, Jerzy Brzostek^2^, Ryszard Konior^3^, Falko G Panzer^4^, Nancy A François^5^, Sudheer M Ravula^6^, Devayani A Kolhe^6^, Yue Song^5^, Ilse Dieussaert^5^, Lode Schuerman^5^, Dorota Borys^5^

**Affiliation:**

^1^Department of Preventive Medicine, Poznan University School of Medical Sciences, Poznan, Poland

^2^Pediatrics Department, Zespol Opieki Zdrowotnej w Debicy, Debica, Poland

^3^Neuro-infection and Pediatric Neurology, John Paul II Hospital, Cracow, Poland

^4^Gemeinschaftspraxis für Kinder- und Jugendmedizin, Mannheim, Germany

^5^Vaccine Research and Development, GSK Vaccines, Wavre, Belgium

^6^GSK Vaccines, Bangalore, India

**Corresponding author:**

Jacek Wysocki

Department of Preventive Medicine
Poznan University of Medical Sciences
ul. Smoluchowskiego 11, 60-179 Poznan

[jwysocki@ump.edu.pl](mailto:jwysocki@ump.edu.pl).

**Keywords:** antibody persistence, immunologic memory, *Streptococcus pneumoniae*, pneumococcal conjugate vaccine, children

**List of abbreviations**: 7vCRM, 7-valent pneumococcal conjugate vaccine; AE, adverse event; ATP, according-to-protocol; CI, confidence interval; DTPa-HBV-IPV/Hib, diphtheria-tetanus-acellular pertussis-hepatitis B-inactivated poliomyelitis and *Haemophilus influenzae* type b vaccine; ELISA, enzyme-linked immunosorbent assay; EL.U, ELISA units; GCP, Good Clinical Practice; GMC, geometric mean concentration; GMT, geometric mean titer; MenC-CV, meningococcal serogroup C conjugate vaccine; OPA, opsonophagocytic activity; PCV, pneumococcal conjugate vaccine; PD, protein D; PHiD-CV, pneumococcal non-typeable *Haemophilus influenzae* protein D conjugate vaccine; SAE, serious adverse event; TVC, total vaccinated cohort.

**Abstract**

To investigate long-term antibody persistence following the administration of the 10-valent pneumococcal non-typeable *Haemophilus influenzae* protein D conjugate vaccine (PHiD-CV), we present results of two follow-up studies assessing antibody persistence following two 3+1 schedules up to four (NCT00624819 – Study A) and five years (NCT00891176 – Study B) post-booster vaccination. In Study A, antibody persistence was measured one, two and four years post-booster in children previously primed and boosted with PHiD-CV, or primed with the 7-valent pneumococcal conjugate vaccine (7vCRM) and boosted with either PHiD-CV or 7vCRM. In Study B, PHiD-CV was co-administered with meningococcal vaccines, and pneumococcal antibody persistence was measured two, three and five years post-booster. An age-matched control group, unvaccinated against *Streptococcus pneumoniae*, was enrolled in Study A, allowing assessment of immunologic memory by administration of one dose of PHiD-CV to both primed (four years post-booster) and unprimed six-year-old children. Four years post-booster (Study A), antibody concentrations and opsonophagocytic activity (OPA) titers remained higher compared to the pre-booster timepoint, with no major differences between the three primed groups. Antibody persistence was also observed in Study B, with minimal differences between groups. The additional PHiD-CV dose administered four years post-booster in Study A elicited more robust immune responses in primed children than in unprimed children. Long-term serotype-specific antibody persistence and robust immunologic memory responses observed in these two studies suggest induction of long-term protection against pneumococcal disease after PHiD-CV vaccination.

**Keywords:** antibody persistence, immunologic memory, *Streptococcus pneumoniae*, pneumococcal conjugate vaccine, children

**Introduction**

*Streptococcus pneumoniae* is a leading cause of bacterial meningitis, sepsis, pneumonia and acute otitis media.[^1^](#_ENREF_1) The greatest disease burden is in children younger than five years.[^2-4^](#_ENREF_2) Antibodies against capsular polysaccharides of various *S. pneumoniae* serotypes provide serotype-specific protection against pneumococcal infections through antibody-mediated opsonophagocytosis,^[5-8](#_ENREF_5)^ hence assessment of long term persistence of antibodies is important. However, no correlate of protection has yet been established.

Previous studies showed that infant vaccination with the 10-valent pneumococcal non-typeable *Haemophilus influenzae* protein D conjugate vaccine (PHiD-CV, *Synflorix™*, GSK Vaccines)[^9^](#_ENREF_9) led to a decrease in the incidence of invasive pneumococcal disease,[^5-7^](#_ENREF_5)^,^ [^10^](#_ENREF_10)^,^ [^11^](#_ENREF_11) acute otitis media and community-acquired pneumonia,[^12-14^](#_ENREF_12) as well as a marked decrease in antibiotic prescriptions.[^15^](#_ENREF_15) PHiD-CV was shown to be immunogenic,[^16^](#_ENREF_16) to induce immunologic memory up to two years post-booster vaccination,[^17^](#_ENREF_17)^,^ [^18^](#_ENREF_18) and to have a clinically acceptable safety profile when co-administered with routine pediatric vaccines.[^19^](#_ENREF_19) However, antibody persistence after infant vaccination with PHiD-CV beyond two years post-booster is unclear.[^17^](#_ENREF_17)^,^ [^18^](#_ENREF_18) Moreover, as mass vaccination schedules expand, there is a risk that adding further antigens to the schedule could lead to unexpected immune interferences.[^20^](#_ENREF_20)^,^ [^21^](#_ENREF_21)

In order to address these concerns, two studies were performed to determine persistence of serotype-specific pneumococcal antibodies and opsonophagocytic activity (OPA) following two 3+1 vaccination schedules up to four and five years post-booster vaccination. In the first study (**Study A**), we assessed antibody persistence at one, two and four years post-booster in children who received pneumococcal conjugate vaccines (PCVs; PHiD-CV or 7vCRM – the licensed 7-valent PCV, *Prevenar™/Prevnar™*, Pfizer) in the first two years of life, and we evaluated immunologic memory four years post-booster. In the second study (**Study B**), we assessed antibody persistence at two, three, and five years post-booster in children who received PHiD-CV or 7vCRM co-administered with meningococcal serogroup C conjugate vaccines (MenC-CVs).

**Results**

The design of the two studies is displayed with vaccine administration and blood sampling schedules in **Figure 1**.

***Demographics***

A total of 524 children vaccinated with PCVs in previous studies[^16^](#_ENREF_16) were enrolled one year post-booster in **Study A**, and 426 completed the four-year follow-up. The according-to-protocol (ATP) cohort for antibody persistence analysis included 523 children one year post-booster, 494 children two years post-booster and 358 children four years post-booster. At year four, parents did not provide consent for participation in the immunologic memory assessment for 77 out of 426 children; thus, only 349 primed children were included in the immunologic memory assessment. In addition, at year four, 100 age-matched children who had not previously received any pneumococcal vaccine (subsequently referred to as “Unprimed”) were enrolled, totaling to 449 children included in the total vaccinated cohort (TVC) for immunologic memory assessment. Out of these, 378 children remained in the ATP cohort for immunologic memory assessment. The number of children in the TVC/ATP cohorts at different timepoints is detailed in **Figure 2**, and demographic characteristics of different study groups are presented in **Table 1**.

In **Study B**, a total of 581, 561 and 539 children who had received PHiD-CV or 7vCRM co-administered with MenC-CVs in previous studies[^22^](#_ENREF_22) were respectively enrolled two, three and five years post-booster, and 539 completed the five-year follow-up. The ATP cohort for antibody persistence analysis included 571 children two years post-booster, 543 children three years post-booster and 530 children five years post-booster. The number of children enrolled in each cohort at different timepoints is shown in **Figure 3**, and demographic characteristics of these cohorts are presented in **Table 1**.

***Antibody persistence in Study A***

In all three primed groups (PHiD-CV, 7vCRM and 7vCRM/PHiD-CV), for each of the vaccine serotypes, a decline in antibody geometric mean concentrations (GMCs, **Figure 4**) and OPA geometric mean titers (GMTs, **Figure 5**) was observed one year post-booster. Two years post-booster, antibody GMCs and OPA GMTs generally remained in the same range as one year post-booster (exceptions were a decrease of antibody GMCs for serotypes 4 and 18C, and an increase for serotypes 14 and 19F). Four years post-booster, antibody GMCs either increased or remained at the same level as year two, except for serotype 4 for which a decrease was observed (**Figure 4**). Few differences could be observed for common serotypes between groups in terms of antibody GMCs and OPA GMTs at all timepoints (**Figures 4** and **5**).

Four years post-booster, the percentage of children with antibody concentrations ≥0.20 µg/mL were similar between the PHiD-CV, 7vCRM and 7vCRM/PHiD-CV groups for the seven common serotypes (except for serotype 4) and serotype 1 (unique to PHiD-CV), regardless of the antibody levels at the previous timepoints. For serotypes 5 and 7F (unique to PHiD-CV), these values appeared to be similar between the PHiD-CV and 7vCRM/PHiD-CV groups (**Table S1**). At the same timepoint, the percentage of children with antibody concentrations ≥0.05 µg/mL were comparable among groups, except for serotype 7F that appeared to be similar between PHiD-CV receiving groups only (**Table S2**). The percentages of children with OPA titers ≥8 were within similar ranges between all groups for most serotypes (**Table S3**).

Although anti-protein D (anti-PD) antibody GMCs were also observed to decline from one month to four years post-booster in the PHiD-CV group, their levels remained higher than those in the 7vCRM group at all timepoints (**Table 2**).

***Antibody persistence in Study B***

For all vaccine serotypes, antibody GMCs declined from one month to two years post-booster (**Figure 6**), then stabilized up to five years post-booster (except for serotype 4 where antibody GMCs continued to decrease in all groups). Similarly, OPA GMTs declined from one month to two years post-booster (**Figure 7**), then stabilized up to three years post-booster, the last timepoint at which OPA GMTs were evaluated. When comparing the four groups, no major differences were observed in terms of antibody GMCs and OPA GMTs for common serotypes at all timepoints, except for consistent trend of higher OPA GMT for serotypes 4, 6B and 23F in 7vCRM/HibMenC-TT group post-booster vaccination (**Figures** **6** and **7**).

In PHiD-CV vaccinees, the percentages of children with antibody concentrations or OPA titers above the predefined thresholds decreased at each subsequent timepoint after the booster dose for most vaccine serotypes, but values remained similar between groups, except for serotype 5 (percentages of children with OPA titers ≥8) and serotype 18C (percentages of children with antibody concentration and OPA titers above thresholds) (**Tables S4** and **S5**).

Data on antibody persistence from **Study A** and **Study B** did not reveal major differences, suggesting that co-administration of MenC-CVs did not alter persistence of immune responses to PHiD-CV.

Antibody GMCs against PD tended to decline with time from the post-booster timepoint in all three groups receiving PHiD-CV, but remained consistently higher than that in the 7vCRM group up to five years post-booster (**Table 3**).

***Immunologic memory assessment by measuring the early immune responses 7–10 days following an additional dose of PHiD-CV four years post-booster in Study A***

For each vaccine and vaccine-related (6A and 19A) serotype, substantial increases in the antibody GMCs and OPA GMTs were observed in all groups 7–10 days post-additional dose, compared to pre-vaccination (**Figure 8**).

For most serotypes, the additional dose elicited more robust immune responses (antibody GMCs and OPA GMTs) to the 10 vaccine pneumococcal serotypes at 7–10 days post-vaccination in the three primed groups compared to those observed in the Unprimed group. This is indicative of an anamnestic immune response in primed children (**Figure 8**). For serotypes 1, 5 and 7F, antibody GMCs at 7–10 days post-vaccination were similar in the PHiD-CV and 7vCRM/PHiD-CV groups (**Figure 8A**).

Anti-PD antibody GMCs were higher at 7–10 days post-vaccination in the PHiD-CV group than in the three other groups, where similar anti-PD antibody GMCs were observed (**Table 2**).

***Safety***

In **Study A**, one serious adverse event (SAE, bronchopneumonia) was reported in one child during the first year of follow-up. It resolved without sequelae and it was considered by the investigator not causally related to the vaccination. In all groups, the additional dose of PHiD-CV administered in six-year-olds was well tolerated and no SAEs were reported.

No SAEs that were considered to be causally related to the vaccination or study participation were reported in **Study B**.

**Discussion**

Vaccine-elicited pneumococcal antibodies have been shown to persist up to four years[^23^](#_ENREF_23) and an anamnestic immune response has been seen five years[^24^](#_ENREF_24) after administration of the 7vCRM vaccine. Here, we observed antibody persistence for all vaccine pneumococcal and vaccine-related serotypes (6A and 19A) four (**Study A**) and five years (**Study B**) after the PHiD-CV booster dose; previous studies assessed antibody persistence up to two years post-booster.[^17^](#_ENREF_17)^,^ [^18^](#_ENREF_18)

As expected, post-booster antibody GMCs and OPA GMTs tended to decrease gradually over time. However, children with declining post-vaccination antibody concentrations and OPA titers do not necessarily become susceptible to pneumococcal disease. In a previous study, an antibody decline was noted after vaccination with 9vCRM, but vaccine efficacy against invasive pneumococcal disease was still 77.8% six years post-vaccination.[^25^](#_ENREF_25) The induction of immunologic memory following vaccination is considered to be a key factor in the long-term protection against invasive pneumococcal disease.

For each of the common vaccine serotypes, antibody GMCs, OPA GMTs and percentages of children with antibody concentrations and OPA titers above the predefined thresholds tended to be similar between primed groups up to four (**Study A**) or five years (**Study B**) post-booster, regardless of antibody levels in the previous years. Antibody persistence was observed against all serotypes analyzed, including those not commonly found in the nasopharynx and for which the limited natural exposure is thus unlikely to have contributed to the persistence of antibodies.[^26^](#_ENREF_26)

The primary mechanism of defense against *S. pneumoniae* is antibody-mediated opsonophagocytosis.[^27^](#_ENREF_27) Our results show that functional antibodies persist up to four years after booster vaccination with PHiD-CV. Limited data on OPA persistence is available in the literature, and we have not found any published reports of persistence of OPA responses on such a long time-scale. Therefore, our findings that there were limited differences between groups in both studies and that the co-administration of MenC-CVs did not affect long-term OPA persistence are of interest.

One of the key features of conjugate vaccines is that they facilitate the induction of immunologic memory and therefore, through the rapid induction of a robust anamnestic response upon subsequent pathogen exposure, contribute to long-term protection against bacterial infections. The induction of immune memory following previous primary and/or booster vaccination with PHiD-CV was assessed in **Study A** through the measurement of early immune responses following the administration of an additional dose of PHiD-CV four years post-booster. Higher antibody GMCs and OPA GMTs in the PHiD-CV primed/boosted children compared to the unprimed children provide evidence of immunologic memory induced by PHiD-CV.

Persistence of anti-PD antibodies has also been observed in both studies. Our findings indicate that a single previous dose of PHiD-CV is not sufficient to prime the immune response, as anti-PD antibody GMCs following administration of an additional PHiD-CV dose four years post-booster were essentially the same in the 7vCRM/PHiD-CV and 7vCRM groups as well as in the Unprimed group. Even though the clinical significance of antibody concentrations against PD has not been established, a previous study has shown that the efficacy of an 11-valent pneumococcal PD-conjugate vaccine against episodes of acute otitis media caused by non-typeable *H. influenzae* was 35.3% (95% CI, 1.8–57.4).[^28^](#_ENREF_28)

The studies presented here have several limitations. Comparison of the results between groups in **Study A** should be done with caution because study objectives were exploratory (no pre-defined criteria and no correction for multiplicity). Also, the small sample size of the 7vCRM group was a potential limitation. A limitation of **Study B** was the lack of assessment of OPA responses five years after the booster dose.

In conclusion, persistence of immune responses induced by PHiD-CV was observed four to five years post-booster in six- and seven-year-old children. Moreover, an additional dose administered in six-year-olds elicited a more robust immune response for all vaccine serotypes and vaccine-related serotypes (6A and 19A), in primed children than in age-matched unprimed children, indicative of an anamnestic response in primed children. These results suggest that PHiD-CV protective efficacy may extend until at least four years after booster vaccination.

**Materials and Methods**

***Study design, vaccines and participants***

**Study A** was a Phase III, open-label, controlled multicenter long-term follow-up study with four groups (**Figure 1**) performed in six centers in Poland between March 2008 and November 2011 (ClinicalTrials.gov; NCT00624819). This study was a continuation of a primary vaccination study (NCT00307554) and a booster study (NCT00370396) performed in Finland, France and Poland.[^16^](#_ENREF_16) The study population at enrollment (one year after the booster dose) was composed of healthy children aged 28–32 months who were previously vaccinated according to a 3+1 schedule at 2, 3, 4 and 12–18 months of age with either PHiD-CV (**PHiD-CV group**), 7vCRM (**7vCRM group**), or 7vCRM primary + PHiD-CV booster (**7vCRM/PHiD-CV group**), co-administered with diphtheria-tetanus-acellular pertussis-hepatitis B-inactivated poliomyelitis and *H. influenzae* type b vaccine ([DTPa-HBV-IPV/Hib], *Infanrix hexa™*, GSK Vaccines).[^16^](#_ENREF_16) For all children, informed consent was obtained at study entry at year one post-booster and an additional Informed Consent Form was acquired for the children involved in the immunologic memory assessment. Anti-pneumococcal IgG antibodies and OPA were measured at one, two, and four years post-booster dose for antibody persistence assessment. For assessment of immunologic memory, an additional dose of PHiD-CV was offered at year four post-booster to each primed child who entered this study phase, and a catch-up dose of PHiD-CV to enrolled age-matched 64–68 month-old healthy children who had not previously received any pneumococcal vaccines (**Unprimed** **group**).

The study vaccine PHiD-CV (0.5 mL per dose) contained 1 µg of each capsular polysaccharide from pneumococcal serotypes 1, 5, 6B, 7F, 9V, 14 and 23F, and 3 µg of serotype 4 conjugated to non-typeable *H. influenzae* PD, 3 µg of capsular polysaccharide of serotype 18C conjugated to tetanus toxoid, and 3 µg of capsular polysaccharide of serotype 19F conjugated to diphtheria toxoid. The vaccine (lot# ASPNA060F) was administered in the deltoid muscle of the participants in **Study A**.

**Study B** was a Phase III, open label, controlled multicenter long-term follow-up study with four parallel groups (**Figure 1**) performed in 24 centers in Germany, Poland and Spain between May 2009 and November 2012 (NCT00891176) as a continuation of a primary vaccination study (NCT00334334) and a booster study (NCT00463437).[^19^](#_ENREF_19)^,^ [^22^](#_ENREF_22)^,^ [^29^](#_ENREF_29) Details of the vaccination schedule, vaccine composition and study groups can be found in **Figure 1**, and have been described previously.[^19^](#_ENREF_19)^,^ [^22^](#_ENREF_22)^,^ [^29^](#_ENREF_29) Briefly, children received 3+1 doses of PHiD-CV or 7vCRM co-administered with MenC-CVs at 2, 4, 6 and 11–18 months of age. PHiD-CV was co-administered with either MenC-CRM_197_ (*Meningitec*^TM^, Nuron Biotech), MenC-TT (*NeisVac-C*^TM^, Pfizer) or HibMenC-TT (*Menitorix*^TM^, GSK Vaccines), while 7vCRM was only co-administered with HibMenC-TT. Anti-pneumococcal IgG antibodies were measured at two, three and five years post-booster dose and OPA responses were determined two and three years post-booster dose for antibody persistence assessment. No study vaccine was administered during this long-term follow-up.

***Immunogenicity assessment***

For the assessment of antibody persistence, blood samples were taken at the previously described timepoints for both studies. For the assessment of immunologic memory in **Study A**, blood samples were taken prior to and 7–10 days after the PHiD-CV dose administered to primed and unprimed six-year-old children. After blood centrifugation and serum separation, samples were stored at or below -20 °C until analysis.

IgG antibodies against 10 pneumococcal vaccine serotypes (1, 4, 5, 6B, 7F, 9V, 14, 18C, 19F, 23F) and 2 vaccine-related serotypes (6A and 19A) were measured by 22F-inhibition enzyme-linked immunosorbent assay (ELISA; in-house assay at GSK Biologicals laboratory or validated laboratory designated by GSK Biologicals), as described previously.[^30^](#_ENREF_30)^,^ [^31^](#_ENREF_31) The percentage of children with antibody concentrations above 0.05 µg/mL and 0.20 µg/mL are presented here. A concentration of 0.20 µg/mL measured using GSK’s 22F-inhibition ELISA assay corresponds to a concentration of 0.35 µg/mL as determined using the World Health Organization reference ELISA without 22F adsorption,[^30^](#_ENREF_30) which is agreed to represent the non-inferiority threshold for licensure of new PCVs for active immunization against invasive pneumococcal disease.

OPA was measured by a killing assay using an HL-60 cell line. The results were presented as the dilution of serum (opsonic titer) able to sustain 50% killing of live pneumococci under assay conditions (in-house assay at a GSK Biologicals laboratory or validated laboratory designated by GSK Biologicals). The cut-off of the assay was an opsonic titer of 8 as previously described.[^32^](#_ENREF_32)

IgG antibodies to the *H. influenzae* PD were measured by an ELISA assay developed by GSK Biologicals, with the nonlipidated PD as coating material. Concentration of specific PD antibodies was determined using a standard reference serum. The cut-off of the assay was 100 ELISA units (EL.U) per milliliter.

***Safety reporting***

Vaccine-related SAEs were to be recorded from the end of the booster vaccination study up to the end of the follow-up studies for **Studies A** and **B.**

In addition, in **Study A**, following administration of PHiD-CV, parents or guardians were asked to complete diary cards to report local (pain, redness and swelling) and general symptoms (fever, irritability/fussiness, drowsiness and loss of appetite) over the first four days after PHiD-CV vaccination, and unsolicited adverse events (AEs) over 31 days after vaccination. Safety data for the unprimed children have been reported previously.[^33^](#_ENREF_33) All SAEs following the PHiD-CV vaccination were to be recorded up to the study end.

***Statistical analysis***

The statistical analyses were performed using the Statistical Analysis System (SAS) Drug and Development (SDD) Web portal version 3.5 and SAS version 9.22.

Immunogenicity analyses were performed based on the ATP cohorts for antibody persistence year one, year two and year four and on the ATP cohort for immunologic memory year four in **Study A** and on the ATP cohorts for antibody persistence year two, year three and year five in **Study B**, which included only children who complied with study procedures and had results available for at least one vaccine antigen.

Antibody GMCs, OPA GMTs and percentages of children reaching predefined immunologic thresholds or cut-offs were calculated with 95% confidence intervals (CIs) for each vaccine pneumococcal serotype and for vaccine-related serotypes 6A and 19A. Anti-PD antibody GMCs and seropositivity rates were also calculated with 95% CIs.

Antibody concentrations and OPA titers below the assay cut-off were given an arbitrary value of half of the cut-off for the purpose of GMC and GMT calculations. The safety analysis was performed based on the TVC.

***Ethics***

The study protocol was approved by ethics review committees of participating centers. Written informed consent was obtained from the parents or legal guardians before enrollment. Overall, the studies were undertaken in accordance with principles of Good Clinical Practice (GCP) guidelines and the Declaration of Helsinki. During the course of the study, whenever potential or actual issues with regard to the conduct of the study were identified, either via site monitoring activities or brought to GSK Biologicals’ attention by other oversight mechanisms, these issues were investigated and appropriate corrective and/or preventive actions were taken.

In **Study A**, obvious incoherence in the immunogenicity results per consecutive timepoints of the same children was detected in three out of six study sites. Based on evidence of a systematic error of mislabelling in two sites, individual serology results in the database between the two timepoints were switched (a total of 110 children). For the third clinical site, no evidence of systematic error was found to explain the incoherence, so the data (obtained from 59 children) from this site were excluded from any immunogenicity analysis.

In **Study B**, some deviations in terms of study documentation and adherence to the study protocol were identified. At one of the study centers some additional GCP deviations were noted such as the finding that annual reports and updates of informed consent had not been sent to regulatory authorities, as required by local law. However, after a full investigation by a GSK department independent of the study team and discussion with local regulatory agencies, it was determined that these findings did not have an impact on the safety of children or on data integrity.

**Disclosure of Potential Conflict of Interest**

JW declares receiving travel grants to participate in international scientific congresses and fees for lecturers from the GSK groups of companies; YS works as a consultant in XPE Pharma & Science for the GSK group of companies; NF, DK, ID, LS and DB are employed by the GSK group of companies; ID, LS and DB own stock options and restricted shares; SR was employed by the GSK group of companies and declares no conflict of interest. Other authors declare no conflict of interest.

**Contributorship section**

LS, DB, ID and NF designed the study. ID, JW, JB, RK and FP acquired the data. NF, DK, SR, ID, DB, JW and YS performed or supervised the analysis. JW, JB, RK and FP contributed to the conduct of the study (monitoring of study participants). All authors participated in the interpretation of the data and all reviewed and approved the final version of the report.

**Acknowledgements**

The authors would like to thank investigators Hanna Czajka, Knecht Roland, Mathias H. Wagner and GSK team member Jacqueline Miller. The authors also acknowledge Mihai Surducan and Ioana Cristina Ilea (XPE Pharma & Science) for medical writing support and Marie-Line Seret and Bram Blomme (XPE Pharma & Science on behalf of GSK) for manuscript coordination.

**Funding**

GlaxoSmithKline Biologicals SA was the funding source and was involved in all stages of the conduct and analysis for both studies. GlaxoSmithKline Biologicals SA also took responsibility for all costs associated with the development and publishing of the present manuscript.

**Trademarks**

Synflorix, Infanrix hexa and Menitorix are trademarks of the GSK group of companies. Prevenar/Prevnar and NeisVac-C are trademarks of Pfizer. Meningitec is a trademark of Nuron Biotech.

**References**

1. Rudan I, O'Brien KL, Nair H, Liu L, Theodoratou E, Qazi S, Luksic I, Fischer Walker CL, Black RE, Campbell H, et al. Epidemiology and etiology of childhood pneumonia in 2010: estimates of incidence, severe morbidity, mortality, underlying risk factors and causative pathogens for 192 countries. J Glob Health 2013; 3:010401.

2. Davis SM, Deloria-Knoll M, Kassa HT, O'Brien KL. Impact of pneumococcal conjugate vaccines on nasopharyngeal carriage and invasive disease among unvaccinated people: review of evidence on indirect effects. Vaccine 2013; 32:133-45.

3. Loo JD, Conklin L, Fleming-Dutra KE, Knoll MD, Park DE, Kirk J, Goldblatt D, O'Brien KL, Whitney CG. Systematic review of the indirect effect of pneumococcal conjugate vaccine dosing schedules on pneumococcal disease and colonization. Pediatr Infect Dis J 2014; 33 Suppl 2:S161-71.

4. Conklin L, Loo JD, Kirk J, Fleming-Dutra KE, Deloria Knoll M, Park DE, Goldblatt D, O'Brien KL, Whitney CG. Systematic review of the effect of pneumococcal conjugate vaccine dosing schedules on vaccine-type invasive pneumococcal disease among young children. Pediatr Infect Dis J 2014; 33 Suppl 2:S109-18.

5. De Wals P, Lefebvre B, Defay F, Deceuninck G, Boulianne N. Invasive pneumococcal diseases in birth cohorts vaccinated with PCV-7 and/or PHiD-CV in the province of Quebec, Canada. Vaccine 2012; 30:6416-20.

6. De Wals P, Lefebvre B, Markowski F, Deceuninck G, Defay F, Douville-Fradet M, Landry M. Impact of 2+1 pneumococcal conjugate vaccine program in the province of Quebec, Canada. Vaccine 2014; 32:1501-6.

7. Palmu AA, Jokinen J, Borys D, Nieminen H, Ruokokoski E, Siira L, Puumalainen T, Lommel P, Hezareh M, Moreira M, et al. Effectiveness of the ten-valent pneumococcal Haemophilus influenzae protein D conjugate vaccine (PHiD-CV10) against invasive pneumococcal disease: a cluster randomised trial. Lancet 2013; 381:214-22.

8. Hausdorff WP, Hoet B, Adegbola RA. Predicting the impact of new pneumococcal conjugate vaccines: serotype composition is not enough. Expert Rev Vaccines 2015; 14:413-28.

9. Croxtall JD, Keating GM. Pneumococcal polysaccharide protein D-conjugate vaccine (Synflorix; PHiD-CV). Paediatr Drugs 2009; 11:349-57.

10. Jokinen J, Rinta-Kokko H, Siira L, Palmu AA, Virtanen MJ, Nohynek H, Virolainen-Julkunen A, Toropainen M, Nuorti JP. Impact of ten-valent pneumococcal conjugate vaccination on invasive pneumococcal disease in Finnish children--a population-based study. PLoS One 2015; 10:e0120290.

11. Domingues CM, Verani JR, Montenegro Renoiner EI, de Cunto Brandileone MC, Flannery B, de Oliveira LH, Santos JB, de Moraes JC, for the Brazilian Pneumococcal Conjugate Vaccine Effectiveness Study Group. Effectiveness of ten-valent pneumococcal conjugate vaccine against invasive pneumococcal disease in Brazil: a matched case-control study. Lancet Respir Med 2014; 2:464-71.

12. Sgambatti S, Minamisava R, Bierrenbach AL, Toscano CM, Vieira MA, Policena G, Andrade AL. Early impact of 10-valent pneumococcal conjugate vaccine in childhood pneumonia hospitalizations using primary data from an active population-based surveillance. Vaccine 2016; 34:663-70.

13. Tregnaghi MW, Sáez-Llorens X, López P, Abate H, Smith E, Pósleman A, Calvo A, Wong D, Cortes-Barbosa C, Ceballos A, et al. Efficacy of pneumococcal nontypable Haemophilus influenzae protein D conjugate vaccine (PHiD-CV) in young Latin American children: a double-blind randomized controlled trial. PLoS Med 2014; 11:e1001657.

14. Afonso ET, Minamisava R, Bierrenbach AL, Escalante JJ, Alencar AP, Domingues CM, Morais-Neto OL, Toscano CM, Andrade AL. Effect of 10-valent pneumococcal vaccine on pneumonia among children, Brazil. Emerg Infect Dis 2013; 19:589-97.

15. Palmu AA, Jokinen J, Nieminen H, Rinta-Kokko H, Ruokokoski E, Puumalainen T, Borys D, Lommel P, Traskine M, Moreira M, et al. Effect of pneumococcal Haemophilus influenzae protein D conjugate vaccine (PHiD-CV10) on outpatient antimicrobial purchases: a double-blind, cluster randomised phase 3-4 trial. Lancet Infect Dis 2014; 14:205-12.

16. Vesikari T, Wysocki J, Chevallier B, Karvonen A, Czajka H, Arsène JP, Lommel P, Dieussaert I, Schuerman L. Immunogenicity of the 10-valent pneumococcal non-typeable Haemophilus influenzae protein D conjugate vaccine (PHiD-CV) compared to the licensed 7vCRM vaccine. Pediatr Infect Dis J 2009; 28:S66-76.

17. Prymula R, Habib A, Francois N, Borys D, Schuerman L. Immunological memory and nasopharyngeal carriage in 4-year-old children previously primed and boosted with 10-valent pneumococcal non-typeable Haemophilus influenzae protein D conjugate vaccine (PHiD-CV) with or without concomitant prophylactic paracetamol. Vaccine 2013; 31:2080-8.

18. Silfverdal SA, Skerlikova H, Zanova M, Papúchová D, Traskine M, Borys D, Schuerman L. Anamnestic immune response in 3- to 4-year-old children previously immunized with 10-valent pneumococcal nontypeable Haemophilus influenzae protein D conjugate vaccine as 2-dose or 3-dose priming and a booster dose in the first year of life. Pediatr Infect Dis J 2011; 30:e155-63.

19. Chevallier B, Vesikari T, Brzostek J, Knuf M, Bermal N, Aristegui J, Borys D, Cleerbout J, Lommel P, Schuerman L. Safety and reactogenicity of the 10-valent pneumococcal non-typeable Haemophilus influenzae protein D conjugate vaccine (PHiD-CV) when coadministered with routine childhood vaccines. Pediatr Infect Dis J 2009; 28:S109-18.

20. Dagan R, Poolman J, Siegrist CA. Glycoconjugate vaccines and immune interference: a review. Vaccine 2010; 28:5513-23.

21. Slack MH, Schapira D, Thwaites RJ, Burrage M, Southern J, Andrews N, Borrow R, Goldblatt D, Miller E. Immune response of premature infants to meningococcal serogroup C and combined diphtheria-tetanus toxoids-acellular pertussis-Haemophilus influenzae type b conjugate vaccines. J Infect Dis 2001; 184:1617-20.

22. Wysocki J, Tejedor JC, Grunert D, Konior R, Garcia-Sicilia J, Knuf M, Bernard L, Dieussaert I, Schuerman L. Immunogenicity of the 10-valent pneumococcal non-typeable Haemophilus influenzae protein D conjugate vaccine (PHiD-CV) when coadministered with different neisseria meningitidis serogroup C conjugate vaccines. Pediatr Infect Dis J 2009; 28:S77-88.

23. Ekström N, Ahman H, Palmu A, Grönholm S, Kilpi T, Käyhty H, for The FinOM Study Group. Concentration and high avidity of pneumococcal antibodies persist at least 4 years after immunization with pneumococcal conjugate vaccine in infancy. Clin Vaccine Immunol 2013; 20:1034-40.

24. Madhi SA, Klugman KP, Kuwanda L, Cutland C, Käyhty H, Adrian P. Quantitative and qualitative anamnestic immune responses to pneumococcal conjugate vaccine in HIV-infected and HIV-uninfected children 5 years after vaccination. J Infect Dis 2009; 199:1168-76.

25. Madhi SA, Adrian P, Kuwanda L, Jassat W, Jones S, Little T, Soininen A, Cutland C, Klugman KP. Long-term immunogenicity and efficacy of a 9-valent conjugate pneumococcal vaccine in human immunodeficient virus infected and non-infected children in the absence of a booster dose of vaccine. Vaccine 2007; 25:2451-7.

26. Simell B, Auranen K, Kayhty H, Goldblatt D, Dagan R, O'Brien KL, for The Pneumococcal Carriage Group. The fundamental link between pneumococcal carriage and disease. Expert Rev Vaccines 2012; 11:841-55.

27. Song JY, Moseley MA, Burton RL, Nahm MH. Pneumococcal vaccine and opsonic pneumococcal antibody. J Infect Chemother 2013; 19:412-25.

28. Prymula R, Peeters P, Chrobok V, Kriz P, Novakova E, Kaliskova E, Kohl I, Lommel P, Poolman J, Prieels JP, et al. Pneumococcal capsular polysaccharides conjugated to protein D for prevention of acute otitis media caused by both Streptococcus pneumoniae and non-typable Haemophilus influenzae: a randomised double-blind efficacy study. Lancet 2006; 367:740-8.

29. Knuf M, Szenborn L, Moro M, Petit C, Bermal N, Bernard L, Dieussaert I, Schuerman L. Immunogenicity of routinely used childhood vaccines when coadministered with the 10-valent pneumococcal non-typeable Haemophilus influenzae protein D conjugate vaccine (PHiD-CV). Pediatr Infect Dis J 2009; 28:S97-S108.

30. Henckaerts I, Goldblatt D, Ashton L, Poolman J. Critical differences between pneumococcal polysaccharide enzyme-linked immunosorbent assays with and without 22F inhibition at low antibody concentrations in pediatric sera. Clin Vaccine Immunol 2006; 13:356-60.

31. Poolman JT, Frasch CE, Käyhty H, Lestrate P, Madhi SA, Henckaerts I. Evaluation of pneumococcal polysaccharide immunoassays using a 22F adsorption step with serum samples from infants vaccinated with conjugate vaccines. Clin Vaccine Immunol 2010; 17:134-42.

32. Henckaerts I, Durant N, De Grave D, Schuerman L, Poolman J. Validation of a routine opsonophagocytosis assay to predict invasive pneumococcal disease efficacy of conjugate vaccine in children. Vaccine 2007; 25:2518-27.

33. Brzostek J, Wysocki J, François N, Ravula S, Song Y, Dieussaert I, Schuerman L, Borys D. Immunogenicity and safety of 2-dose catch-up vaccination with the 10-valent pneumococcal non-typeable *Haemophilus influenzae* protein D conjugate vaccine (PHiD-CV) in the 6th year of life. Presented at: 32th Annual Meeting of the European Society for Paediatric Infectious Diseases; 2014 May 6-10; Dublin, Ireland.

**Figure Legends**

**Figure 1. Study procedures for Study A and Study B**

**Notes**: In **Study A**, all doses of PCVs were co-administered with DTPa-HBV-IPV/Hib at primary and booster vaccination (Finland, Poland and France), except for the 2nd dose in France that was co-administered with DTPa-IPV/Hib. In **Study B**, the PHiD-CV/MenC-CRM and PHiD-CV/MenC-TT groups received: the meningococcal vaccines as 2-dose primary vaccination in Germany and Spain, 3-dose primary vaccination in Poland (3rd dose received after blood sampling); PHiD-CV was co-administered with DTPa-HBV-IPV/Hib at primary vaccination and DTPa-HBV-IPV/Hib (Germany and Poland) or DTPa-IPV/Hib (Spain) at booster vaccination. In the PHiD-CV/HibMenC-TT and 7vCRM/HibMenC-TT groups, PCVs were co-administered with DTPa-HBV-IPV at primary vaccination and with DTPa-HBV-IPV (Germany and Poland) or DTPa-IPV (Spain) at booster vaccination.

^$^Blood sample collected for immunologic memory assessment 7–10 days after the PHiD-CV dose at Y4, in **Study A**; Y=number of years following booster vaccination in PCV-vaccinated children; Pri=primary; Bst=booster; PCV=pneumococcal conjugate vaccine.

**Figure 2. Flow diagram for Study A.**

N=number of children; Y=number of years following booster vaccination in PCV-vaccinated children; TVC=total vaccinated cohort; ATP=according-to-protocol cohort; PHiD-CV=children previously primed and boosted with PHiD-CV; 7vCRM=children previously primed and boosted with 7vCRM; 7vCRM/PHiD-CV=children previously primed with 7vCRM and boosted with PHiD-CV; Unprimed=age-matched children not previously vaccinated with any pneumococcal vaccine and enrolled as control group for the immunologic memory assessment.

**Figure 3. Flow diagram for Study B.**

N=number of children; Y=number of years following booster vaccination; ATP=according-to-protocol cohort. PHiD-CV/MenC-CRM=children receiving PHiD-CV co-administered with MenC-CRM; PHiD-CV/MenC-TT=children receiving PHiD-CV co-administered with MenC-TT; PHiD-CV/HibMenC-TT=children receiving PHiD-CV co-administered with HibMenC-TT; 7vCRM/HibMenC-TT=children receiving 7vCRM co-administered with HibMenC-TT.

**Figure 4. Serotype-specific pneumococcal antibody GMCs following primary and booster vaccination up to year 4 post-booster (Study A) (ATP cohort for immunogenicity and ATP cohorts for antibody persistence at Y1, Y2 and Y4, respectively)**

*vaccine-related serotypes; GMC=geometric mean concentration; ATP=according-to-protocol; Y=number of years following booster vaccination; N=maximum number of children with available results at primary vaccination/booster vaccination/Y1/Y2/Y4; pre-pri=before the 1st dose of primary vaccination; post-pri=1 month after the 3rd dose of primary vaccination; pre-bst=before the booster dose; post-bst=1 month after the booster dose; error bars indicate 95% confidence intervals.

**Figure 5. Serotype-specific pneumococcal OPA GMTs post-primary and post-booster vaccination up to year 4 post-booster (Study A) (ATP cohort for immunogenicity and ATP cohorts for antibody persistence at Y1, Y2 and Y4, respectively)**

*vaccine-related serotypes; OPA=opsonophagocytic activity; GMT=geometric mean titer; ATP=according-to-protocol; Y=number of years following booster vaccination; N=maximum number of children with available results at primary vaccination/booster vaccination/Y1/Y2/Y4; post-pri=1 month after the 3rd dose of primary vaccination; pre-bst=before the booster dose; post-bst=1 month after the booster dose; error bars indicate 95% confidence intervals.

**Figure 6. Serotype-specific pneumococcal antibody GMCs post-primary and post-booster vaccination up to year 5 post-booster (Study B) (ATP cohort for immunogenicity and ATP cohorts for antibody persistence at Y2, Y3 and Y5, respectively)**

GMC=geometric mean concentration; ATP=according-to-protocol; Y=number of years following booster vaccination; N=maximum number of children with available results at primary vaccination/booster vaccination/Y2/Y3/Y5; post-pri=1 month after the 3rd dose of primary vaccination; pre-bst=before the booster dose; post-bst=1 month after the booster dose; error bars indicate 95% confidence intervals.

**Figure 7. Serotype-specific pneumococcal OPA GMTs post-primary and post-booster vaccination up to year 3 post-booster (Study B) (ATP cohort for immunogenicity and ATP cohorts for antibody persistence at Y2 and Y3, respectively)**

*testing of *Streptococcus pneumoniae* opsonophagocytic activity (OPA) was not performed at year 5; GMT=geometric mean titer; ATP=according-to-protocol; Y=number of years following booster vaccination; N=maximum number of children with available results at primary vaccination/booster vaccination/Y2/Y3; post-pri=1 month after the 3rd dose of primary vaccination; pre-bst=before the booster dose; post-bst=1 month after the booster dose; error bars indicate 95% confidence intervals.

**Figure 8. Serotype-specific pneumococcal antibody GMCs (A) and OPA GMTs (B) before and 7–10 days after the additional PHiD-CV** **dose in PCV-vaccinated children or the first PHiD-CV** **dose in the Unprimed group (Study A) (ATP cohort for immunologic memory at year 4)**

*vaccine-related serotypes; PCV=pneumococcal conjugate vaccine; ATP=according-to-protocol; GMC=geometric mean concentration; OPA=opsonophagocytic activity; GMT=geometric mean titer; N=maximum number of children with available results; pre=before the additional PHiD-CV dose in PCV-vaccinated children or before the first PHiD-CV dose in the Unprimed group; post=7–10 days after the additional PHiD-CV dose in PCV-vaccinated children or 7–10 days after the first PHiD-CV dose in the Unprimed group; error bars indicate 95% confidence intervals.
