# Supplementary material for: Antibody persistence and immunologic memory in children vaccinated with 4 doses of pneumococcal conjugate vaccines: Results from 2 long-term follow-up studies
Source: Hum Vaccin Immunother. 2016 Oct 13;13(3):661–75. doi: 10.1080/21645515.2016.1241919 (PMC5360132; doi:10.1080/21645515.2016.1241919)
Supplement: KHVI_A_1241919_Supplementary_material.zip [file khvi-13-03-1241919-s001.zip › 2016HV0225R-s03.docx]

**Table 1. Demographic characteristics of the study population by timepoint (ATP cohorts for the respective timepoints) (Study A and Study B)**

| Study A |  | PHiD-CV |  | 7vCRM |  | 7vCRM/PHiD-CV |  | Unprimed |
| --- | --- | --- | --- | --- | --- | --- | --- | --- |
|  |  |  |  |  |  |  |  |  |
| *ATP cohorts for antibody persistence Y1* |  |  |  |  |  |  |  |  |
| N |  | 390 |  | 31 |  | 102 |  | - |
| Age (months)/Mean±SD |  | 29.1±0.87 |  | 29.0±0.75 |  | 29.1±0.81 |  | - |
| Sex (female) n (%) |  | 203 (52.1) |  | 14 (45.2) |  | 52 (51.0) |  | - |
| *ATP cohorts for antibody persistence Y2* | | |  |  |  |  |  |  |
| N |  | 368 |  | 30 |  | 96 |  | - |
| Age (months)/Mean±SD |  | 41.1±1.03 |  | 41.0±0.83 |  | 41.2±1.04 |  | - |
| Sex (female) n (%) |  | 191 (51.9) |  | 14 (46.7) |  | 50 (52.1) |  | - |
| *ATP cohorts for antibody persistence Y4* | | | | | | | | |
| N |  | 264 |  | 19 |  | 75 |  | - |
| Age (months)/Mean±SD |  | 67.4±0.79 |  | 67.3±1.00 |  | 67.4±0.84 |  | - |
| Sex (female) n (%) |  | 140 (53.0) |  | 7 (36.8) |  | 43 (57.3) |  | - |
| *ATP cohorts for immunologic memory Y4* | | | | | | | | |
| N |  | 211 |  | 14 |  | 55 |  | 98 |
| Age (months)/Mean±SD |  | 67.3±0.83 |  | 67.1±1.07 |  | 67.2±0.90 |  | 65.4±1.30 |
| Sex (female) n (%) |  | 112 (53.1) |  | 5 (35.7) |  | 30 (54.5) |  | 53 (54.1) |
| Study B |  | PHiD-CV/MenC-CRM |  | PHiD-CV/MenC-TT |  | PHiD-CV/HibMenC-TT |  | 7vCRM/HibMenC-TT |
| *ATP cohorts for antibody persistence Y2* |  |  |  |  |  |  |  |  |
| N |  | 141 |  | 146 |  | 144 |  | 140 |
| Age (months)/Mean±SD |  | 37.1±1.17 |  | 37.2±1.21 |  | 37.3±1.18 |  | 37.3±1.28 |
| Sex (female) n (%) |  | 71 (50.4) |  | 65 (44.5) |  | 83 (57.6) |  | 71 (50.7) |
| *ATP cohorts for antibody persistence Y3* | | | | | | | | |
| N |  | 136 |  | 138 |  | 133 |  | 136 |
| Age (months)/Mean±SD |  | 48.8±1.25 |  | 48.7±1.13 |  | 49.2±1.53 |  | 48.7±1.22 |
| Sex (female) n (%) |  | 69 (50.7) |  | 60 (43.5) |  | 80 (60.2) |  | 71 (52.2) |
| *ATP cohorts for antibody persistence Y5* | | | | | | | | |
| N |  | 128 |  | 137 |  | 131 |  | 134 |
| Age (months)/Mean±SD |  | 72.8±1.02 |  | 72.9±1.09 |  | 72.9±1.00 |  | 72.9±1.15 |
| Sex (female) n (%) |  | 63 (49.2) |  | 63 (46.0) |  | 77 (58.8) |  | 68 (50.7) |

N=number of children; SD=standard deviation; n (%)=number (percentage) of children in a given category; ATP=according-to-protocol; Y=number of years following booster vaccination in PCV-vaccinated children.

**Table 2. Seropositivity rates and GMCs for anti-protein D antibodies by timepoint (ATP cohorts for the respective timepoints) (Study A)**

| Timepoint |  | PHiD-CV | |  | 7vCRM | |  | 7vCRM/PHiD-CV | |  | Unprimed | |
| --- | --- | --- | --- | --- | --- | --- | --- | --- | --- | --- | --- | --- |
|  |  | N | % ≥100 EL.U/mL,  (LL; UL) |  | N | % ≥100 EL.U/mL,  (LL; UL) |  | N | % ≥100 EL.U/mL,  (LL; UL) |  | N | % ≥100 EL.U/mL,  (LL; UL) |
| *ATP cohorts for immunogenicity at primary/booster vaccination* | | | | | | | | | | | | |
| Post-pri |  | 1095 | 99.8 (99.3; 100) |  | 364 | 17.9 (14.1; 22.2) |  | 364 | 17.9 (14.1; 22.2) |  | - | - |
| Pre-bst |  | 338 | 94.7 (91.7; 96.8) |  | 73 | 20.5 (12.0; 31.6) |  | 127 | 28.3 (20.7; 37.0) |  | - | - |
| Post-bst |  | 340 | 99.4 (97.9; 99.9) |  | 86 | 18.6 (11.0; 28.4) |  | 134 | 50.0 (41.2; 58.8) |  | - | - |
| *ATP cohorts for antibody persistence* | | | | | | | | | | | | |
| Y1 |  | 390 | 95.6 (93.1; 97.4) |  | 30 | 36.7 (19.9; 56.1) |  | 102 | 69.6 (59.7; 78.3) |  | - | - |
| Y2 |  | 368 | 92.4 (89.2; 94.9) |  | 29 | 51.7 (32.5; 70.6) |  | 96 | 67.7 (57.4; 76.9) |  | - | - |
| Y4 |  | 261 | 92.3 (88.4; 95.3) |  | 19 | 63.2 (38.4; 83.7) |  | 74 | 73.0 (61.4; 82.6) |  | - | - |
| *ATP cohorts for immunologic memory Y4* | | | |  |  |  |  |  |  |  |  |  |
| Pre |  | 208 | 91.3 (86.7; 94.8) |  | 14 | 64.3 (35.1; 87.2) |  | 54 | 66.7 (52.5; 78.9) |  | 95 | 56.8 (46.3; 67.0) |
| D7-10-Post |  | 208 | 99.5 (97.4; 100) |  | 14 | 100 (76.8; 100) |  | 54 | 100 (93.4; 100) |  | 96 | 95.8 (89.7; 98.9) |
|  |  | N | GMC, EL.U/mL  (LL; UL) |  | N | GMC, EL.U/mL  (LL; UL) |  | N | GMC, EL.U/mL  (LL; UL) |  | N | GMC, EL.U/mL  (LL; UL) |
| *ATP cohorts for immunogenicity at primary/booster vaccination* | | | | | | | | | | | | |
| Post-pri |  | 1095 | 1529.9 (1452.3; 1611.8) |  | 364 | 66.1 (61.6; 70.9) |  | 364 | 66.1 (61.6; 70.9) |  | - | - |
| Pre-bst |  | 338 | 556.4 (494.7; 625.7) |  | 73 | 72.3 (59.3; 88.0) |  | 127 | 78.1 (67.8; 89.9) |  | - | - |
| Post-bst |  | 340 | 2887.6 (2573.7; 3239.8) |  | 86 | 75.3 (60.0; 94.4) |  | 134 | 125.5 (103.4; 152.4) |  | - | - |
| *ATP cohorts for antibody persistence* | | | | | | |  |  |  |  |  |  |
| Y1 |  | 390 | 822.1 (731.5; 923.9) |  | 30 | 93.9 (66.6; 132.3) |  | 102 | 193.6 (155.9; 240.4) |  | - | - |
| Y2 |  | 368 | 573.2 (509.6; 644.8) |  | 29 | 116.7 (80.9; 168.3) |  | 96 | 157.5 (128.7; 192.8) |  | - | - |
| Y4 |  | 261 | 372.4 (329.6; 420.9) |  | 19 | 144.9 (86.3; 243.2) |  | 74 | 161.4 (128.4; 203.0) |  | - | - |
| *ATP cohorts for immunologic memory Y4* | | | |  |  |  |  |  |  |  |  |  |
| Pre |  | 208 | 374.3 (324.8; 431.3) |  | 14 | 133.3 (78.3; 227.0) |  | 54 | 141.6 (109.1; 183.8) |  | 95 | 106.0 (91.1; 123.4) |
| D7-10-Post |  | 208 | 2106.0 (1806.7; 2454.9) |  | 14 | 718.2 (442.5; 1165.7) |  | 54 | 680.7 (522.9; 886.2) |  | 96 | 382.9 (320.7; 457.2) |

GMC=geometric mean antibody concentration; ATP=according-to-protocol; EL.U=ELISA units; % ≥100 EL.U/mL=percentage of children with antibody concentration ≥ 100 EL.U/mL; N=number of children with available results; LL=lower limit of the 95% confidence interval; UL=upper limit of the 95% confidence interval; post-pri=1 month after the 3rd dose of primary vaccination; pre-bst=before the booster dose; post-bst=1 month after the booster dose; Y=number of years following booster vaccination in PCV-vaccinated children; pre=before the additional PHiD-CV dose in PCV-vaccinated children or before the first PHiD-CV dose in the Unprimed group; D7-10 post=7–10 days after the additional dose in PCV-vaccinated children or 7–10 days after the first dose in the Unprimed group.

**Table 3. Seropositivity rates and GMCs for anti-protein D antibodies by timepoint (ATP cohorts for the respective timepoints) (Study B)**

| Timepoint |  | PHiD-CV/MenC-CRM | |  | PHiD-CV/MenC-TT | |  | PHiD-CV/HibMenC-TT | |  | 7vCRM/HibMenC-TT | |
| --- | --- | --- | --- | --- | --- | --- | --- | --- | --- | --- | --- | --- |
|  |  | N | % ≥100 EL.U/mL,  (LL; UL) |  | N | % ≥100 EL.U/mL,  (LL; UL) |  | N | % ≥100 EL.U/mL,  (LL; UL) |  | N | % ≥100 EL.U/mL,  (LL; UL) |
| *ATP cohorts for immunogenicity at primary/booster vaccination* | | | | | | | | | | | | |
| Post-pri |  | 168 | 100 (97.8; 100) |  | 174 | 100 (97.9; 100) |  | 173 | 99.4 (96.8; 100) |  | 163 | 23.3 (17.1; 30.6) |
| Pre-bst |  | 147 | 98.0 (94.2; 99.6) |  | 153 | 96.1 (91.7; 98.5) |  | 147 | 93.9 (88.7; 97.2) |  | 146 | 40.4 (32.4; 48.8) |
| Post-bst |  | 158 | 100 (97.7; 100) |  | 152 | 99.3 (96.4; 100) |  | 160 | 100 (97.7; 100) |  | 148 | 44.6 (36.4; 53.0) |
| *ATP cohorts for antibody persistence* | | | | | | | | | | | | |
| Y2 |  | 140 | 94.3 (89.1; 97.5) |  | 145 | 94.5 (89.4; 97.6) |  | 144 | 94.4 (89.3; 97.6) |  | 139 | 49.6 (41.1; 58.2) |
| Y3 |  | 136 | 93.4 (87.8; 96.9) |  | 138 | 92.8 (87.1; 96.5) |  | 129 | 89.1 (82.5; 93.9) |  | 132 | 56.8 (47.9; 65.4) |
| Y5 |  | 128 | 88.3 (81.4; 93.3) |  | 137 | 86.9 (80.0; 92.0) |  | 131 | 86.3 (79.2; 91.6) |  | 133 | 55.6 (46.8; 64.2) |
|  |  | N | GMC, EL.U/mL (LL; UL) |  | N | GMC, EL.U/mL (LL; UL) |  | N | GMC, EL.U/mL (LL; UL) |  | N | GMC, EL.U/mL (LL; UL) |
| *ATP cohorts for immunogenicity at primary/booster vaccination* | | | | | | | | | | | | |
| Post-pri |  | 168 | 2114.0 (1847.6; 2418.8) |  | 174 | 1715.5 (1494.9; 1968.7) |  | 173 | 1726.7 (1493.3; 1996.7) |  | 163 | 72.3 (64.5; 81.1) |
| Pre-bst |  | 147 | 779.2 (665.2; 912.8) |  | 153 | 666.6 (563.2; 789.1) |  | 147 | 635.8 (529.9; 763.0) |  | 146 | 96.0 (82.9; 111.1) |
| Post-bst |  | 158 | 3106.0 (2693.8; 3581.3) |  | 152 | 2598.4 (2206.9; 3059.4) |  | 160 | 2679.3 (2305.5; 3113.6) |  | 148 | 96.4 (84.1; 110.5) |
| *ATP cohorts for antibody persistence* | | | | | | | | | | | | |
| Y2 |  | 140 | 494.6 (414.0; 591.0) |  | 145 | 444.8 (373.2; 530.1) |  | 144 | 439.8 (366.4; 527.9) |  | 139 | 100.3 (87.6; 114.9) |
| Y3 |  | 136 | 401.1 (338.3; 475.6) |  | 138 | 387.2 (326.7; 458.9) |  | 129 | 334.8 (277.7; 403.7) |  | 132 | 107.9 (94.6; 123.1) |
| Y5 |  | 128 | 284.7 (239.5; 338.4) |  | 137 | 278.0 (236.3; 327.1) |  | 131 | 266.1 (222.2; 318.8) |  | 133 | 105.2 (92.4; 119.8) |

GMC=geometric mean antibody concentration; ATP=according-to-protocol; EL.U=ELISA units; % ≥100 EL.U/mL=percentage of children with antibody concentration ≥ 100 EL.U/mL; N=number of children with available results; LL=lower limit of the 95% confidence interval; UL=upper limit of the 95% confidence interval; post-pri=1 month after the 3rd dose of primary vaccination; pre-bst=before the booster dose; post-bst=1 month after the booster dose; Y=number of years following booster vaccination.
