# Supplementary material for: Antibody persistence and immunologic memory in children vaccinated with 4 doses of pneumococcal conjugate vaccines: Results from 2 long-term follow-up studies
Source: Hum Vaccin Immunother. 2016 Oct 13;13(3):661–75. doi: 10.1080/21645515.2016.1241919 (PMC5360132; doi:10.1080/21645515.2016.1241919)
Supplement: KHVI_A_1241919_Supplementary_material.zip [file khvi-13-03-1241919-s001.zip › 2016HV0225R-s04.docx]

**Antibody persistence and immunologic memory in children vaccinated with four doses of pneumococcal conjugate vaccines: results from two long-term follow-up studies**

By Jacek WYSOCKI *et al.*

**Supplementary tables.**

**Table S1. Percentages of children with serotype-specific pneumococcal antibody concentrations ≥ 0.20 µg/mL by timepoint (ATP cohorts for the respective timepoints) (Study A)**

| Serotype | Timepoint | **PHiD-CV** | |  | **7vCRM** | |  | **7vCRM/PHiD-CV** | |
| --- | --- | --- | --- | --- | --- | --- | --- | --- | --- |
|  |  | N | % (LL; UL) |  | N | % (LL; UL) |  | N | % (LL; UL) |
| **1** | Pre-pri | 1102 | 8.0 (6.5; 9.7) |  | 375 | 5.9 (3.7; 8.7) |  | 375 | 5.9 (3.7; 8.7) |
|  | Post-pri | 1100 | 97.3 (96.1; 98.2) |  | 371 | 4.0 (2.3; 6.6) |  | 371 | 4.0 (2.3; 6.6) |
|  | Pre-bst | 338 | 36.4 (31.3; 41.8) |  | 82 | 3.7 (0.8; 10.3) |  | 133 | 2.3 (0.5; 6.5) |
|  | Post-bst | 342 | 99.4 (97.9; 99.9) |  | 81 | 4.9 (1.4; 12.2) |  | 133 | 85.0 (77.7; 90.6) |
|  | Y1 | 387 | 65.4 (60.4; 70.1) |  | 29 | 10.3 (2.2; 27.4) |  | 102 | 60.8 (50.6; 70.3) |
|  | Y2 | 367 | 48.5 (43.3; 53.7) |  | 29 | 10.3 (2.2; 27.4) |  | 96 | 39.6 (29.7; 50.1) |
|  | Y4 | 263 | 46.0 (39.9; 52.2) |  | 19 | 42.1 (20.3; 66.5) |  | 75 | 48.0 (36.3; 59.8) |
| **4** | Pre-pri | 1101 | 6.8 (5.4; 8.5) |  | 372 | 7.8 (5.3; 11.0) |  | 372 | 7.8 (5.3; 11.0) |
|  | Post-pri | 1106 | 97.1 (95.9; 98.0) |  | 373 | 100 (99.0; 100) |  | 373 | 100 (99.0; 100) |
|  | Pre-bst | 342 | 57.3 (51.9; 62.6) |  | 78 | 67.9 (56.4; 78.1) |  | 131 | 75.6 (67.3; 82.7) |
|  | Post-bst | 343 | 99.7 (98.4; 100) |  | 88 | 100 (95.9; 100) |  | 133 | 100 (97.3; 100) |
|  | Y1 | 387 | 84.5 (80.5; 88.0) |  | 31 | 100 (88.8; 100) |  | 102 | 97.1 (91.6; 99.4) |
|  | Y2 | 368 | 56.0 (50.7; 61.1) |  | 30 | 66.7 (47.2; 82.7) |  | 96 | 81.3 (72.0; 88.5) |
|  | Y4 | 263 | 47.1 (41.0; 53.4) |  | 19 | 63.2 (38.4; 83.7) |  | 74 | 71.6 (59.9; 81.5) |
| **5** | Pre-pri | 1099 | 8.0 (6.5; 9.8) |  | 375 | 8.3 (5.7; 11.5) |  | 375 | 8.3 (5.7; 11.5) |
|  | Post-pri | 1104 | 99.0 (98.2; 99.5) |  | 374 | 1.9 (0.8; 3.8) |  | 374 | 1.9 (0.8; 3.8) |
|  | Pre-bst | 344 | 67.2 (61.9; 72.1) |  | 84 | 6.0 (2.0; 13.3) |  | 134 | 10.4 (5.8; 16.9) |
|  | Post-bst | 342 | 99.4 (97.9; 99.9) |  | 82 | 6.1 (2.0; 13.7) |  | 133 | 85.7 (78.6; 91.2) |
|  | Y1 | 387 | 87.6 (83.9; 90.7) |  | 30 | 13.3 (3.8; 30.7) |  | 101 | 79.2 (70.0; 86.6) |
|  | Y2 | 368 | 77.4 (72.8; 81.6) |  | 29 | 24.1 (10.3; 43.5) |  | 95 | 68.4 (58.1; 77.6) |
|  | Y4 | 263 | 71.9 (66.0; 77.2) |  | 19 | 36.8 (16.3; 61.6) |  | 74 | 73.0 (61.4; 82.6) |
| **6B** | Pre-pri | 1102 | 19.6 (17.3; 22.1) |  | 374 | 21.4 (17.3; 25.9) |  | 374 | 21.4 (17.3; 25.9) |
|  | Post-pri | 1100 | 65.9 (63.0; 68.7) |  | 372 | 79.0 (74.5; 83.1) |  | 372 | 79.0 (74.5; 83.1) |
|  | Pre-bst | 333 | 67.0 (61.6; 72.0) |  | 75 | 30.7 (20.5; 42.4) |  | 131 | 52.7 (43.8; 61.5) |
|  | Post-bst | 341 | 96.5 (93.9; 98.2) |  | 87 | 97.7 (91.9; 99.7) |  | 133 | 98.5 (94.7; 99.8) |
|  | Y1 | 389 | 76.3 (71.8; 80.5) |  | 31 | 93.5 (78.6; 99.2) |  | 102 | 82.4 (73.6; 89.2) |
|  | Y2 | 368 | 71.2 (66.3; 75.8) |  | 30 | 86.7 (69.3; 96.2) |  | 96 | 75.0 (65.1; 83.3) |
|  | Y4 | 263 | 88.2 (83.7; 91.8) |  | 19 | 89.5 (66.9; 98.7) |  | 75 | 88.0 (78.4; 94.4) |
| **7F** | Pre-pri | 1102 | 27.9 (25.3; 30.7) |  | 375 | 26.1 (21.8; 30.9) |  | 375 | 26.1 (21.8; 30.9) |
|  | Post-pri | 1107 | 99.5 (98.8; 99.8) |  | 375 | 4.5 (2.7; 7.2) |  | 375 | 4.5 (2.7; 7.2) |
|  | Pre-bst | 340 | 90.6 (87.0; 93.5) |  | 85 | 4.7 (1.3; 11.6) |  | 133 | 2.3 (0.5; 6.5) |
|  | Post-bst | 342 | 100 (98.9; 100) |  | 85 | 7.1 (2.6; 14.7) |  | 133 | 95.5 (90.4; 98.3) |
|  | Y1 | 387 | 94.3 (91.5; 96.4) |  | 31 | 25.8 (11.9; 44.6) |  | 102 | 96.1 (90.3; 98.9) |
|  | Y2 | 368 | 89.4 (85.8; 92.4) |  | 30 | 26.7 (12.3; 45.9) |  | 96 | 87.5 (79.2; 93.4) |
|  | Y4 | 263 | 80.2 (74.9; 84.9) |  | 19 | 47.4 (24.4; 71.1) |  | 75 | 85.3 (75.3; 92.4) |
| **9V** | Pre-pri | 1098 | 16.3 (14.2; 18.6) |  | 375 | 17.6 (13.9; 21.8) |  | 375 | 17.6 (13.9; 21.8) |
|  | Post-pri | 1103 | 98.1 (97.1; 98.8) |  | 374 | 99.5 (98.1; 99.9) |  | 374 | 99.5 (98.1; 99.9) |
|  | Pre-bst | 344 | 84.6 (80.3; 88.2) |  | 77 | 90.9 (82.2; 96.3) |  | 130 | 94.6 (89.2; 97.8) |
|  | Post-bst | 340 | 100 (98.9; 100) |  | 89 | 100 (95.9; 100) |  | 133 | 100 (97.3; 100) |
|  | Y1 | 388 | 94.3 (91.5; 96.4) |  | 31 | 100 (88.8; 100) |  | 102 | 93.1 (86.4; 97.2) |
|  | Y2 | 368 | 81.5 (77.2; 85.4) |  | 30 | 86.7 (69.3; 96.2) |  | 96 | 74.0 (64.0; 82.4) |
|  | Y4 | 263 | 77.2 (71.6; 82.1) |  | 19 | 73.7 (48.8; 90.9) |  | 75 | 76.0 (64.7; 85.1) |
| **14** | Pre-pri | 1097 | 64.3 (61.3; 67.1) |  | 374 | 67.9 (62.9; 72.6) |  | 374 | 67.9 (62.9; 72.6) |
|  | Post-pri | 1100 | 99.5 (98.9; 99.9) |  | 374 | 99.5 (98.1; 99.9) |  | 374 | 99.5 (98.1; 99.9) |
|  | Pre-bst | 336 | 79.8 (75.1; 83.9) |  | 75 | 93.3 (85.1; 97.8) |  | 130 | 96.2 (91.3; 98.7) |
|  | Post-bst | 339 | 99.1 (97.4; 99.8) |  | 86 | 100 (95.8; 100) |  | 133 | 100 (97.3; 100) |
|  | Y1 | 388 | 95.6 (93.1; 97.4) |  | 31 | 100 (88.8; 100) |  | 102 | 96.1 (90.3; 98.9) |
|  | Y2 | 368 | 94.0 (91.1; 96.2) |  | 30 | 96.7 (82.8; 99.9) |  | 96 | 95.8 (89.7; 98.9) |
|  | Y4 | 263 | 97.0 (94.1; 98.7) |  | 19 | 100 (82.4; 100) |  | 75 | 97.3 (90.7; 99.7) |
| **18C** | Pre-pri | 1096 | 28.7 (26.1; 31.5) |  | 375 | 27.2 (22.8; 32.0) |  | 375 | 27.2 (22.8; 32.0) |
|  | Post-pri | 1102 | 96.0 (94.7; 97.1) |  | 374 | 98.9 (97.3; 99.7) |  | 374 | 98.9 (97.3; 99.7) |
|  | Pre-bst | 341 | 70.4 (65.2; 75.2) |  | 83 | 72.3 (61.4; 81.6) |  | 131 | 81.7 (74.0; 87.9) |
|  | Post-bst | 343 | 100 (98.9; 100) |  | 87 | 100 (95.8; 100) |  | 134 | 99.3 (95.9; 100) |
|  | Y1 | 384 | 94.8 (92.1; 96.8) |  | 31 | 100 (88.8; 100) |  | 102 | 94.1 (87.6; 97.8) |
|  | Y2 | 368 | 82.1 (77.8; 85.8) |  | 30 | 90.0 (73.5; 97.9) |  | 96 | 80.2 (70.8; 87.6) |
|  | Y4 | 263 | 79.5 (74.1; 84.2) |  | 19 | 78.9 (54.4; 93.9) |  | 74 | 79.7 (68.8; 88.2) |
| **19F** | Pre-pri | 1095 | 49.1 (46.1; 52.1) |  | 374 | 47.3 (42.2; 52.5) |  | 374 | 47.3 (42.2; 52.5) |
|  | Post-pri | 1104 | 95.4 (94.0; 96.5) |  | 375 | 99.2 (97.7; 99.8) |  | 375 | 99.2 (97.7; 99.8) |
|  | Pre-bst | 347 | 78.4 (73.7; 82.6) |  | 85 | 44.7 (33.9; 55.9) |  | 134 | 56.7 (47.9; 65.2) |
|  | Post-bst | 343 | 99.4 (97.9; 99.9) |  | 87 | 100 (95.8; 100) |  | 134 | 97.8 (93.6; 99.5) |
|  | Y1 | 387 | 97.9 (96.0; 99.1) |  | 30 | 90.0 (73.5; 97.9) |  | 102 | 96.1 (90.3; 98.9) |
|  | Y2 | 368 | 91.6 (88.3; 94.2) |  | 30 | 80.0 (61.4; 92.3) |  | 95 | 92.6 (85.4; 97.0) |
|  | Y4 | 263 | 92.8 (88.9; 95.6) |  | 19 | 94.7 (74.0; 99.9) |  | 75 | 100 (95.2; 100) |
| **23F** | Pre-pri | 1098 | 28.1 (25.4; 30.8) |  | 374 | 25.9 (21.6; 30.7) |  | 374 | 25.9 (21.6; 30.7) |
|  | Post-pri | 1102 | 81.4 (79.0; 83.7) |  | 374 | 94.1 (91.2; 96.3) |  | 374 | 94.1 (91.2; 96.3) |
|  | Pre-bst | 338 | 60.9 (55.5; 66.2) |  | 77 | 55.8 (44.1; 67.2) |  | 130 | 75.4 (67.1; 82.5) |
|  | Post-bst | 341 | 97.4 (95.0; 98.8) |  | 88 | 98.9 (93.8; 100) |  | 132 | 97.0 (92.4; 99.2) |
|  | Y1 | 388 | 84.3 (80.3; 87.8) |  | 31 | 100 (88.8; 100) |  | 102 | 92.2 (85.1; 96.6) |
|  | Y2 | 368 | 73.6 (68.8; 78.1) |  | 30 | 96.7 (82.8; 99.9) |  | 96 | 78.1 (68.5; 85.9) |
|  | Y4 | 262 | 83.2 (78.1; 87.5) |  | 19 | 100 (82.4; 100) |  | 75 | 86.7 (76.8; 93.4) |
| **6A*** | Pre-pri | 279 | 21.1 (16.5; 26.4) |  | 93 | 23.7 (15.5; 33.6) |  | 93 | 23.7 (15.5; 33.6) |
|  | Post-pri | 279 | 22.2 (17.5; 27.6) |  | 93 | 31.2 (22.0; 41.6) |  | 93 | 31.2 (22.0; 41.6) |
|  | Pre-bst | 338 | 32.5 (27.6; 37.8) |  | 86 | 23.3 (14.8; 33.6) |  | 133 | 29.3 (21.8; 37.8) |
|  | Post-bst | 340 | 84.4 (80.1; 88.1) |  | 86 | 89.5 (81.1; 95.1) |  | 134 | 77.6 (69.6; 84.4) |
|  | Y1 | 390 | 50.0 (44.9; 55.1) |  | 31 | 67.7 (48.6; 83.3) |  | 102 | 55.9 (45.7; 65.7) |
|  | Y2 | 368 | 53.3 (48.0; 58.5) |  | 30 | 66.7 (47.2; 82.7) |  | 96 | 53.1 (42.7; 63.4) |
|  | Y4 | 263 | 85.2 (80.3; 89.2) |  | 19 | 73.7 (48.8; 90.9) |  | 74 | 86.5 (76.5; 93.3) |
| **19A*** | Pre-pri | 277 | 37.5 (31.8; 43.5) |  | 93 | 49.5 (38.9; 60.0) |  | 93 | 49.5 (38.9; 60.0) |
|  | Post-pri | 279 | 22.6 (17.8; 27.9) |  | 94 | 28.7 (19.9; 39.0) |  | 94 | 28.7 (19.9; 39.0) |
|  | Pre-bst | 340 | 35.0 (29.9; 40.3) |  | 79 | 15.2 (8.1; 25.0) |  | 133 | 24.8 (17.7; 33.0) |
|  | Post-bst | 333 | 83.8 (79.4; 87.6) |  | 81 | 76.5 (65.8; 85.2) |  | 134 | 79.1 (71.2; 85.6) |
|  | Y1 | 390 | 60.5 (55.5; 65.4) |  | 31 | 48.4 (30.2; 66.9) |  | 102 | 46.1 (36.2; 56.2) |
|  | Y2 | 368 | 63.9 (58.7; 68.8) |  | 30 | 56.7 (37.4; 74.5) |  | 96 | 59.4 (48.9; 69.3) |
|  | Y4 | 263 | 90.5 (86.3; 93.8) |  | 19 | 89.5 (66.9; 98.7) |  | 75 | 85.3 (75.3; 92.4) |

*vaccine-related serotypes; ATP=according-to-protocol; N=number of children with available results; LL=lower limit of the 95% confidence interval; UL=upper limit of the 95% confidence interval; pre-pri=before the 1st dose of primary vaccination; post-pri=1 month after the 3rd dose of primary vaccination; pre-bst=before the booster dose; post-bst=1 month after the booster dose; Y=number of years following booster vaccination.

**Table S2. Percentages of children with serotype-specific pneumococcal antibody concentrations ≥ 0.05 µg/mL by timepoint (ATP cohorts for the respective timepoints) (Study A)**

| Serotype | Timepoint | **PHiD-CV** | |  | **7vCRM** | |  | **7vCRM/PHiD-CV** | |
| --- | --- | --- | --- | --- | --- | --- | --- | --- | --- |
|  |  | N | % (LL; UL) |  | N | % (LL; UL) |  | N | % (LL; UL) |
| **1** | Pre-pri | 1102 | 25.9 (23.3; 28.6) |  | 375 | 24.5 (20.3; 29.2) |  | 375 | 24.5 (20.3; 29.2) |
|  | Post-pri | 1100 | 100 (99.7; 100) |  | 371 | 10.0 (7.1; 13.5) |  | 371 | 10.0 (7.1; 13.5) |
|  | Pre-bst | 338 | 89.3 (85.6; 92.4) |  | 82 | 17.1 (9.7; 27.0) |  | 133 | 17.3 (11.3; 24.8) |
|  | Post-bst | 342 | 100 (98.9; 100) |  | 81 | 28.4 (18.9; 39.5) |  | 133 | 98.5 (94.7; 99.8) |
|  | Y1 | 387 | 96.1 (93.7; 97.8) |  | 29 | 24.1 (10.3; 43.5) |  | 102 | 97.1 (91.6; 99.4) |
|  | Y2 | 367 | 96.5 (94.0; 98.1) |  | 29 | 62.1 (42.3; 79.3) |  | 96 | 93.8 (86.9; 97.7) |
|  | Y4 | 263 | 90.5 (86.3; 93.8) |  | 19 | 73.7 (48.8; 90.9) |  | 75 | 94.7 (86.9; 98.5) |
| **4** | Pre-pri | 1101 | 25.1 (22.5; 27.7) |  | 372 | 25.0 (20.7; 29.7) |  | 372 | 25.0 (20.7; 29.7) |
|  | Post-pri | 1106 | 99.8 (99.3; 100) |  | 373 | 100 (99.0; 100) |  | 373 | 100 (99.0; 100) |
|  | Pre-bst | 342 | 96.2 (93.6; 98.0) |  | 78 | 100 (95.4; 100) |  | 131 | 98.5 (94.6; 99.8) |
|  | Post-bst | 343 | 100 (98.9; 100) |  | 88 | 100 (95.9; 100) |  | 133 | 100 (97.3; 100) |
|  | Y1 | 387 | 99.2 (97.8; 99.8) |  | 31 | 100 (88.8; 100) |  | 102 | 100 (96.4; 100) |
|  | Y2 | 368 | 96.7 (94.4; 98.3) |  | 30 | 100 (88.4; 100) |  | 96 | 100 (96.2; 100) |
|  | Y4 | 263 | 90.5 (86.3; 93.8) |  | 19 | 94.7 (74.0; 99.9) |  | 74 | 97.3 (90.6; 99.7) |
| **5** | Pre-pri | 1099 | 41.9 (38.9; 44.8) |  | 375 | 42.4 (37.3; 47.6) |  | 375 | 42.4 (37.3; 47.6) |
|  | Post-pri | 1104 | 100 (99.7; 100) |  | 374 | 17.4 (13.7; 21.6) |  | 374 | 17.4 (13.7; 21.6) |
|  | Pre-bst | 344 | 97.4 (95.1; 98.8) |  | 84 | 36.9 (26.6; 48.1) |  | 134 | 39.6 (31.2; 48.4) |
|  | Post-bst | 342 | 100 (98.9; 100) |  | 82 | 48.8 (37.6; 60.1) |  | 133 | 98.5 (94.7; 99.8) |
|  | Y1 | 387 | 99.7 (98.6; 100) |  | 30 | 63.3 (43.9; 80.1) |  | 101 | 100 (96.4; 100) |
|  | Y2 | 368 | 98.4 (96.5; 99.4) |  | 29 | 72.4 (52.8; 87.3) |  | 95 | 100 (96.2; 100) |
|  | Y4 | 263 | 97.7 (95.1; 99.2) |  | 19 | 100 (82.4; 100) |  | 74 | 98.6 (92.7; 100) |
| **6B** | Pre-pri | 1102 | 48.9 (45.9; 51.9) |  | 374 | 49.5 (44.3; 54.7) |  | 374 | 49.5 (44.3; 54.7) |
|  | Post-pri | 1100 | 88.1 (86.0; 89.9) |  | 372 | 94.9 (92.1; 96.9) |  | 372 | 94.9 (92.1; 96.9) |
|  | Pre-bst | 333 | 94.9 (92.0; 97.0) |  | 75 | 85.3 (75.3; 92.4) |  | 131 | 91.6 (85.5; 95.7) |
|  | Post-bst | 341 | 98.5 (96.6; 99.5) |  | 87 | 98.9 (93.8; 100) |  | 133 | 99.2 (95.9; 100) |
|  | Y1 | 389 | 98.7 (97.0; 99.6) |  | 31 | 100 (88.8; 100) |  | 102 | 98.0 (93.1; 99.8) |
|  | Y2 | 368 | 96.5 (94.0; 98.1) |  | 30 | 100 (88.4; 100) |  | 96 | 99.0 (94.3; 100) |
|  | Y4 | 263 | 98.1 (95.6; 99.4) |  | 19 | 100 (82.4; 100) |  | 75 | 98.7 (92.8; 100) |
| **7F** | Pre-pri | 1102 | 59.9 (56.9; 62.8) |  | 375 | 59.5 (54.3; 64.5) |  | 375 | 59.5 (54.3; 64.5) |
|  | Post-pri | 1107 | 99.9 (99.5; 100) |  | 375 | 26.4 (22.0; 31.2) |  | 375 | 26.4 (22.0; 31.2) |
|  | Pre-bst | 340 | 99.4 (97.9; 99.9) |  | 85 | 14.1 (7.5; 23.4) |  | 133 | 13.5 (8.2; 20.5) |
|  | Post-bst | 342 | 100 (98.9; 100) |  | 85 | 16.5 (9.3; 26.1) |  | 133 | 97.7 (93.5; 99.5) |
|  | Y1 | 387 | 100 (99.1; 100) |  | 31 | 54.8 (36.0; 72.7) |  | 102 | 100 (96.4; 100) |
|  | Y2 | 368 | 99.7 (98.5; 100) |  | 30 | 63.3 (43.9; 80.1) |  | 96 | 99.0 (94.3; 100) |
|  | Y4 | 263 | 98.9 (96.7; 99.8) |  | 19 | 68.4 (43.4; 87.4) |  | 75 | 97.3 (90.7; 99.7) |
| **9V** | Pre-pri | 1098 | 43.4 (40.5; 46.4) |  | 375 | 44.8 (39.7; 50.0) |  | 375 | 44.8 (39.7; 50.0) |
|  | Post-pri | 1103 | 99.9 (99.5; 100) |  | 374 | 99.7 (98.5; 100) |  | 374 | 99.7 (98.5; 100) |
|  | Pre-bst | 344 | 99.1 (97.5; 99.8) |  | 77 | 98.7 (93.0; 100) |  | 130 | 100 (97.2; 100) |
|  | Post-bst | 340 | 100 (98.9; 100) |  | 89 | 100 (95.9; 100) |  | 133 | 100 (97.3; 100) |
|  | Y1 | 388 | 100 (99.1; 100) |  | 31 | 100 (88.8; 100) |  | 102 | 100 (96.4; 100) |
|  | Y2 | 368 | 98.4 (96.5; 99.4) |  | 30 | 100 (88.4; 100) |  | 96 | 100 (96.2; 100) |
|  | Y4 | 263 | 97.0 (94.1; 98.7) |  | 19 | 100 (82.4; 100) |  | 75 | 96.0 (88.8; 99.2) |
| **14** | Pre-pri | 1097 | 89.7 (87.7; 91.4) |  | 374 | 91.2 (87.8; 93.8) |  | 374 | 91.2 (87.8; 93.8) |
|  | Post-pri | 1100 | 99.9 (99.5; 100) |  | 374 | 100 (99.0; 100) |  | 374 | 100 (99.0; 100) |
|  | Pre-bst | 336 | 96.7 (94.2; 98.4) |  | 75 | 98.7 (92.8; 100) |  | 130 | 99.2 (95.8; 100) |
|  | Post-bst | 339 | 100 (98.9; 100) |  | 86 | 100 (95.8; 100) |  | 133 | 100 (97.3; 100) |
|  | Y1 | 388 | 99.7 (98.6; 100) |  | 31 | 100 (88.8; 100) |  | 102 | 100 (96.4; 100) |
|  | Y2 | 368 | 99.7 (98.5; 100) |  | 30 | 100 (88.4; 100) |  | 96 | 100 (96.2; 100) |
|  | Y4 | 263 | 100 (98.6; 100) |  | 19 | 100 (82.4; 100) |  | 75 | 100 (95.2; 100) |
| **18C** | Pre-pri | 1096 | 60.0 (57.1; 63.0) |  | 375 | 61.9 (56.7; 66.8) |  | 375 | 61.9 (56.7; 66.8) |
|  | Post-pri | 1102 | 99.4 (98.7; 99.7) |  | 374 | 99.5 (98.1; 99.9) |  | 374 | 99.5 (98.1; 99.9) |
|  | Pre-bst | 341 | 98.2 (96.2; 99.4) |  | 83 | 97.6 (91.6; 99.7) |  | 131 | 99.2 (95.8; 100) |
|  | Post-bst | 343 | 100 (98.9; 100) |  | 87 | 100 (95.8; 100) |  | 134 | 99.3 (95.9; 100) |
|  | Y1 | 384 | 100 (99.0; 100) |  | 31 | 100 (88.8; 100) |  | 102 | 100 (96.4; 100) |
|  | Y2 | 368 | 99.2 (97.6; 99.8) |  | 30 | 100 (88.4; 100) |  | 96 | 100 (96.2; 100) |
|  | Y4 | 263 | 97.3 (94.6; 98.9) |  | 19 | 100 (82.4; 100) |  | 74 | 98.6 (92.7; 100) |
| **19F** | Pre-pri | 1095 | 78.7 (76.2; 81.1) |  | 374 | 77.3 (72.7; 81.4) |  | 374 | 77.3 (72.7; 81.4) |
|  | Post-pri | 1104 | 98.8 (98.0; 99.4) |  | 375 | 99.7 (98.5; 100) |  | 375 | 99.7 (98.5; 100) |
|  | Pre-bst | 347 | 97.4 (95.1; 98.8) |  | 85 | 96.5 (90.0; 99.3) |  | 134 | 94.8 (89.5; 97.9) |
|  | Post-bst | 343 | 100 (98.9; 100) |  | 87 | 100 (95.8; 100) |  | 134 | 100 (97.3; 100) |
|  | Y1 | 387 | 100 (99.1; 100) |  | 30 | 100 (88.4; 100) |  | 102 | 100 (96.4; 100) |
|  | Y2 | 368 | 100 (99.0; 100) |  | 30 | 100 (88.4; 100) |  | 95 | 100 (96.2; 100) |
|  | Y4 | 263 | 98.9 (96.7; 99.8) |  | 19 | 100 (82.4; 100) |  | 75 | 100 (95.2; 100) |
| **23F** | Pre-pri | 1098 | 55.8 (52.8; 58.8) |  | 374 | 54.0 (48.8; 59.1) |  | 374 | 54.0 (48.8; 59.1) |
|  | Post-pri | 1102 | 95.2 (93.8; 96.4) |  | 374 | 97.6 (95.5; 98.9) |  | 374 | 97.6 (95.5; 98.9) |
|  | Pre-bst | 338 | 90.2 (86.6; 93.2) |  | 77 | 97.4 (90.9; 99.7) |  | 130 | 96.2 (91.3; 98.7) |
|  | Post-bst | 341 | 99.1 (97.5; 99.8) |  | 88 | 98.9 (93.8; 100) |  | 132 | 98.5 (94.6; 99.8) |
|  | Y1 | 388 | 99.5 (98.2; 99.9) |  | 31 | 100 (88.8; 100) |  | 102 | 100 (96.4; 100) |
|  | Y2 | 368 | 97.0 (94.7; 98.5) |  | 30 | 100 (88.4; 100) |  | 96 | 99.0 (94.3; 100) |
|  | Y4 | 262 | 96.9 (94.1; 98.7) |  | 19 | 100 (82.4; 100) |  | 75 | 98.7 (92.8; 100) |
| **6A*** | Pre-pri | 279 | 51.3 (45.2; 57.3) |  | 93 | 60.2 (49.5; 70.2) |  | 93 | 60.2 (49.5; 70.2) |
|  | Post-pri | 279 | 55.9 (49.9; 61.8) |  | 93 | 64.5 (53.9; 74.2) |  | 93 | 64.5 (53.9; 74.2) |
|  | Pre-bst | 338 | 79.3 (74.6; 83.5) |  | 86 | 66.3 (55.3; 76.1) |  | 133 | 72.2 (63.7; 79.6) |
|  | Post-bst | 340 | 95.3 (92.5; 97.3) |  | 86 | 97.7 (91.9; 99.7) |  | 134 | 96.3 (91.5; 98.8) |
|  | Y1 | 390 | 96.4 (94.1; 98.0) |  | 31 | 100 (88.8; 100) |  | 102 | 95.1 (88.9; 98.4) |
|  | Y2 | 368 | 89.7 (86.1; 92.6) |  | 30 | 100 (88.4; 100) |  | 96 | 89.6 (81.7; 94.9) |
|  | Y4 | 263 | 97.3 (94.6; 98.9) |  | 19 | 94.7 (74.0; 99.9) |  | 74 | 97.3 (90.6; 99.7) |
| **19A*** | Pre-pri | 277 | 73.6 (68.0; 78.7) |  | 93 | 84.9 (76.0; 91.5) |  | 93 | 84.9 (76.0; 91.5) |
|  | Post-pri | 279 | 65.2 (59.3; 70.8) |  | 94 | 83.0 (73.8; 89.9) |  | 94 | 83.0 (73.8; 89.9) |
|  | Pre-bst | 340 | 85.6 (81.4; 89.1) |  | 79 | 72.2 (60.9; 81.7) |  | 133 | 69.2 (60.6; 76.9) |
|  | Post-bst | 333 | 98.2 (96.1; 99.3) |  | 81 | 97.5 (91.4; 99.7) |  | 134 | 100 (97.3; 100) |
|  | Y1 | 390 | 93.8 (91.0; 96.0) |  | 31 | 93.5 (78.6; 99.2) |  | 102 | 91.2 (83.9; 95.9) |
|  | Y2 | 368 | 86.7 (82.8; 90.0) |  | 30 | 83.3 (65.3; 94.4) |  | 96 | 86.5 (78.0; 92.6) |
|  | Y4 | 263 | 98.9 (96.7; 99.8) |  | 19 | 94.7 (74.0; 99.9) |  | 75 | 98.7 (92.8; 100) |

*vaccine-related serotypes; ATP=according-to-protocol; N=number of children with available results; LL=lower limit of the 95% confidence interval; UL=upper limit of the 95% confidence interval; pre-pri=before the 1st dose of primary vaccination; post-pri=1 month after the 3rd dose of primary vaccination; pre-bst=before the booster dose; post-bst=1 month after the booster dose; Y=number of years following booster vaccination.

**Table S3. Percentages of children with serotype-specific pneumococcal OPA titers ≥ 8 by timepoint (ATP cohorts for the respective timepoints) (Study A)**

| Serotype | Timepoint | **PHiD-CV** | |  | **7vCRM** | |  | **7vCRM/PHiD-CV** | |
| --- | --- | --- | --- | --- | --- | --- | --- | --- | --- |
|  |  | N | % (LL; UL) |  | N | % (LL; UL) |  | N | % (LL; UL) |
| **1** | Post-pri | 268 | 65.7 (59.7; 71.3) |  | 89 | 4.5 (1.2; 11.1) |  | 89 | 4.5 (1.2; 11.1) |
|  | Pre-bst | 326 | 16.3 (12.4; 20.7) |  | 83 | 6.0 (2.0; 13.5) |  | 126 | 5.6 (2.3; 11.1) |
|  | Post-bst | 301 | 91.0 (87.2; 94.0) |  | 83 | 3.6 (0.8; 10.2) |  | 121 | 31.4 (23.3; 40.5) |
|  | Y1 | 354 | 53.1 (47.8; 58.4) |  | 27 | 7.4 (0.9; 24.3) |  | 92 | 28.3 (19.4; 38.6) |
|  | Y2 | 354 | 26.0 (21.5; 30.9) |  | 27 | 0.0 (0.0; 12.8) |  | 92 | 16.3 (9.4; 25.5) |
|  | Y4 | 250 | 22.0 (17.0; 27.7) |  | 16 | 6.3 (0.2; 30.2) |  | 72 | 23.6 (14.4; 35.1) |
| **4** | Post-pri | 267 | 99.6 (97.9; 100) |  | 88 | 100 (95.9; 100) |  | 88 | 100 (95.9; 100) |
|  | Pre-bst | 295 | 47.8 (42.0; 53.7) |  | 79 | 51.9 (40.4; 63.3) |  | 112 | 60.7 (51.0; 69.8) |
|  | Post-bst | 297 | 100 (98.8; 100) |  | 81 | 100 (95.5; 100) |  | 123 | 100 (97.0; 100) |
|  | Y1 | 340 | 59.1 (53.7; 64.4) |  | 26 | 80.8 (60.6; 93.4) |  | 86 | 75.6 (65.1; 84.2) |
|  | Y2 | 335 | 43.6 (38.2; 49.1) |  | 27 | 44.4 (25.5; 64.7) |  | 84 | 57.1 (45.9; 67.9) |
|  | Y4 | 232 | 46.1 (39.6; 52.8) |  | 16 | 50.0 (24.7; 75.3) |  | 63 | 52.4 (39.4; 65.1) |
| **5** | Post-pri | 263 | 90.9 (86.7; 94.1) |  | 88 | 3.4 (0.7; 9.6) |  | 88 | 3.4 (0.7; 9.6) |
|  | Pre-bst | 305 | 34.4 (29.1; 40.1) |  | 79 | 3.8 (0.8; 10.7) |  | 124 | 1.6 (0.2; 5.7) |
|  | Post-bst | 299 | 96.3 (93.5; 98.1) |  | 83 | 1.2 (0.0; 6.5) |  | 122 | 36.9 (28.3; 46.1) |
|  | Y1 | 335 | 71.9 (66.8; 76.7) |  | 27 | 7.4 (0.9; 24.3) |  | 89 | 51.7 (40.8; 62.4) |
|  | Y2 | 351 | 48.1 (42.8; 53.5) |  | 27 | 7.4 (0.9; 24.3) |  | 91 | 34.1 (24.5; 44.7) |
|  | Y4 | 243 | 32.9 (27.0; 39.2) |  | 15 | 13.3 (1.7; 40.5) |  | 68 | 16.2 (8.4; 27.1) |
| **6B** | Post-pri | 262 | 92.4 (88.5; 95.3) |  | 89 | 95.5 (88.9; 98.8) |  | 89 | 95.5 (88.9; 98.8) |
|  | Pre-bst | 284 | 58.8 (52.8; 64.6) |  | 75 | 53.3 (41.4; 64.9) |  | 120 | 50.0 (40.7; 59.3) |
|  | Post-bst | 295 | 96.6 (93.9; 98.4) |  | 79 | 98.7 (93.1; 100) |  | 118 | 94.9 (89.3; 98.1) |
|  | Y1 | 354 | 46.3 (41.0; 51.7) |  | 25 | 92.0 (74.0; 99.0) |  | 88 | 50.0 (39.1; 60.9) |
|  | Y2 | 342 | 70.5 (65.3; 75.3) |  | 27 | 88.9 (70.8; 97.6) |  | 88 | 76.1 (65.9; 84.6) |
|  | Y4 | 250 | 89.2 (84.7; 92.8) |  | 16 | 93.8 (69.8; 99.8) |  | 68 | 91.2 (81.8; 96.7) |
| **7F** | Post-pri | 264 | 99.6 (97.9; 100) |  | 88 | 18.2 (10.8; 27.8) |  | 88 | 18.2 (10.8; 27.8) |
|  | Pre-bst | 294 | 78.9 (73.8; 83.4) |  | 76 | 36.8 (26.1; 48.7) |  | 117 | 32.5 (24.1; 41.8) |
|  | Post-bst | 296 | 99.7 (98.1; 100) |  | 74 | 31.1 (20.8; 42.9) |  | 118 | 98.3 (94.0; 99.8) |
|  | Y1 | 346 | 99.1 (97.5; 99.8) |  | 25 | 80.0 (59.3; 93.2) |  | 89 | 95.5 (88.9; 98.8) |
|  | Y2 | 350 | 100 (99.0; 100) |  | 27 | 96.3 (81.0; 99.9) |  | 91 | 98.9 (94.0; 100) |
|  | Y4 | 249 | 99.6 (97.8; 100) |  | 15 | 100 (78.2; 100) |  | 71 | 100 (94.9; 100) |
| **9V** | Post-pri | 268 | 100 (98.6; 100) |  | 89 | 100 (95.9; 100) |  | 89 | 100 (95.9; 100) |
|  | Pre-bst | 309 | 97.1 (94.5; 98.7) |  | 81 | 96.3 (89.6; 99.2) |  | 122 | 99.2 (95.5; 100) |
|  | Post-bst | 297 | 100 (98.8; 100) |  | 79 | 100 (95.4; 100) |  | 120 | 100 (97.0; 100) |
|  | Y1 | 352 | 99.7 (98.4; 100) |  | 26 | 100 (86.8; 100) |  | 94 | 95.7 (89.5; 98.8) |
|  | Y2 | 349 | 99.4 (97.9; 99.9) |  | 27 | 100 (87.2; 100) |  | 92 | 94.6 (87.8; 98.2) |
|  | Y4 | 250 | 96.8 (93.8; 98.6) |  | 15 | 93.3 (68.1; 99.8) |  | 70 | 97.1 (90.1; 99.7) |
| **14** | Post-pri | 267 | 99.6 (97.9; 100) |  | 89 | 98.9 (93.9; 100) |  | 89 | 98.9 (93.9; 100) |
|  | Pre-bst | 299 | 82.9 (78.2; 87.0) |  | 79 | 83.5 (73.5; 90.9) |  | 118 | 94.9 (89.3; 98.1) |
|  | Post-bst | 304 | 100 (98.8; 100) |  | 82 | 100 (95.6; 100) |  | 123 | 100 (97.0; 100) |
|  | Y1 | 343 | 98.0 (95.8; 99.2) |  | 25 | 100 (86.3; 100) |  | 91 | 97.8 (92.3; 99.7) |
|  | Y2 | 341 | 97.7 (95.4; 99.0) |  | 25 | 96.0 (79.6; 99.9) |  | 90 | 94.4 (87.5; 98.2) |
|  | Y4 | 250 | 98.8 (96.5; 99.8) |  | 15 | 93.3 (68.1; 99.8) |  | 71 | 98.6 (92.4; 100) |
| **18C** | Post-pri | 266 | 93.6 (90.0; 96.2) |  | 88 | 95.5 (88.8; 98.7) |  | 88 | 95.5 (88.8; 98.7) |
|  | Pre-bst | 309 | 29.4 (24.4; 34.9) |  | 82 | 34.1 (24.0; 45.4) |  | 121 | 28.9 (21.0; 37.9) |
|  | Post-bst | 299 | 99.7 (98.2; 100) |  | 76 | 100 (95.3; 100) |  | 121 | 98.3 (94.2; 99.8) |
|  | Y1 | 319 | 54.2 (48.6; 59.8) |  | 24 | 41.7 (22.1; 63.4) |  | 84 | 29.8 (20.3; 40.7) |
|  | Y2 | 325 | 50.8 (45.2; 56.3) |  | 27 | 40.7 (22.4; 61.2) |  | 83 | 34.9 (24.8; 46.2) |
|  | Y4 | 226 | 53.5 (46.8; 60.2) |  | 16 | 50.0 (24.7; 75.3) |  | 63 | 42.9 (30.5; 56.0) |
| **19F** | Post-pri | 268 | 87.7 (83.1; 91.4) |  | 89 | 92.1 (84.5; 96.8) |  | 89 | 92.1 (84.5; 96.8) |
|  | Pre-bst | 317 | 45.7 (40.2; 51.4) |  | 81 | 14.8 (7.9; 24.4) |  | 123 | 17.9 (11.6; 25.8) |
|  | Post-bst | 293 | 94.9 (91.7; 97.1) |  | 80 | 92.5 (84.4; 97.2) |  | 120 | 98.3 (94.1; 99.8) |
|  | Y1 | 359 | 85.2 (81.1; 88.7) |  | 27 | 70.4 (49.8; 86.2) |  | 93 | 89.2 (81.1; 94.7) |
|  | Y2 | 349 | 79.9 (75.4; 84.0) |  | 27 | 77.8 (57.7; 91.4) |  | 92 | 84.8 (75.8; 91.4) |
|  | Y4 | 247 | 85.4 (80.4; 89.6) |  | 16 | 87.5 (61.7; 98.4) |  | 69 | 89.9 (80.2; 95.8) |
| **23F** | Post-pri | 261 | 93.9 (90.2; 96.5) |  | 87 | 97.7 (91.9; 99.7) |  | 87 | 97.7 (91.9; 99.7) |
|  | Pre-bst | 305 | 70.8 (65.4; 75.9) |  | 77 | 74.0 (62.8; 83.4) |  | 120 | 85.0 (77.3; 90.9) |
|  | Post-bst | 301 | 99.7 (98.2; 100) |  | 80 | 98.8 (93.2; 100) |  | 122 | 98.4 (94.2; 99.8) |
|  | Y1 | 336 | 89.6 (85.8; 92.6) |  | 27 | 96.3 (81.0; 99.9) |  | 93 | 88.2 (79.8; 93.9) |
|  | Y2 | 342 | 83.3 (79.0; 87.1) |  | 26 | 96.2 (80.4; 99.9) |  | 91 | 79.1 (69.3; 86.9) |
|  | Y4 | 239 | 87.4 (82.6; 91.4) |  | 15 | 100 (78.2; 100) |  | 65 | 84.6 (73.5; 92.4) |
| **6A*** | Post-pri | 257 | 58.0 (51.7; 64.1) |  | 89 | 68.5 (57.8; 78.0) |  | 89 | 68.5 (57.8; 78.0) |
|  | Pre-bst | 285 | 55.4 (49.5; 61.3) |  | 77 | 50.6 (39.0; 62.2) |  | 104 | 48.1 (38.2; 58.1) |
|  | Post-bst | 287 | 85.0 (80.4; 88.9) |  | 79 | 94.9 (87.5; 98.6) |  | 113 | 73.5 (64.3; 81.3) |
|  | Y1 | 323 | 60.7 (55.1; 66.0) |  | 25 | 96.0 (79.6; 99.9) |  | 75 | 60.0 (48.0; 71.1) |
|  | Y2 | 314 | 74.8 (69.7; 79.5) |  | 25 | 84.0 (63.9; 95.5) |  | 82 | 75.6 (64.9; 84.4) |
|  | Y4 | 231 | 84.0 (78.6; 88.5) |  | 14 | 85.7 (57.2; 98.2) |  | 66 | 77.3 (65.3; 86.7) |
| **19A*** | Post-pri | 260 | 19.6 (15.0; 25.0) |  | 89 | 3.4 (0.7; 9.5) |  | 89 | 3.4 (0.7; 9.5) |
|  | Pre-bst | 327 | 4.9 (2.8; 7.8) |  | 84 | 2.4 (0.3; 8.3) |  | 126 | 4.8 (1.8; 10.1) |
|  | Post-bst | 287 | 48.8 (42.9; 54.7) |  | 76 | 27.6 (18.0; 39.1) |  | 121 | 23.1 (16.0; 31.7) |
|  | Y1 | 352 | 12.8 (9.5; 16.7) |  | 26 | 11.5 (2.4; 30.2) |  | 93 | 10.8 (5.3; 18.9) |
|  | Y2 | 351 | 32.8 (27.9; 37.9) |  | 27 | 29.6 (13.8; 50.2) |  | 91 | 31.9 (22.5; 42.5) |
|  | Y4 | 246 | 54.9 (48.4; 61.2) |  | 15 | 46.7 (21.3; 73.4) |  | 68 | 50.0 (37.6; 62.4) |

*vaccine-related serotypes; OPA=opsonophagocytic activity; ATP=according-to-protocol; N=number of children with available results; LL=lower limit of the 95% confidence interval; UL=upper limit of the 95% confidence interval; post-pri=1 month after the 3rd dose of primary vaccination; pre-bst=before the booster dose; post-bst=1 month after the booster dose; Y=number of years following booster vaccination.

**Table S4. Percentages of children with serotype-specific pneumococcal antibody concentrations ≥ 0.20 µg/mL by timepoint (ATP cohorts for the respective timepoints) (Study B)**

| Sero-type | Timepoint | **PHiD-CV/MenC-CRM** | |  | **PHiD-CV/MenC-TT** | |  | **PHiD-CV/HibMenC-TT** | |  | **7vCRM/HibMenC-TT** | |
| --- | --- | --- | --- | --- | --- | --- | --- | --- | --- | --- | --- | --- |
|  |  | N | % (LL; UL) |  | N | % (LL; UL) |  | N | % (LL; UL) |  | N | % (LL; UL) |
| **1** | Post-pri | 169 | 96.4 (92.4; 98.7) |  | 174 | 97.7 (94.2; 99.4) |  | 173 | 93.1 (88.2; 96.4) |  | 170 | 0.6 (0.0; 3.2) |
|  | Pre-bst | 150 | 55.3 (47.0; 63.4) |  | 152 | 59.2 (51.0; 67.1) |  | 149 | 55.7 (47.3; 63.8) |  | 151 | 2.6 (0.7; 6.6) |
|  | Post-bst | 158 | 100 (97.7; 100) |  | 152 | 100 (97.6; 100) |  | 160 | 100 (97.7; 100) |  | 151 | 2.6 (0.7; 6.6) |
|  | Y2 | 139 | 63.3 (54.7; 71.3) |  | 146 | 52.7 (44.3; 61.1) |  | 142 | 50.7 (42.2; 59.2) |  | 139 | 17.3 (11.4; 24.6) |
|  | Y3 | 136 | 52.9 (44.2; 61.6) |  | 138 | 47.1 (38.6; 55.8) |  | 131 | 48.9 (40.0; 57.7) |  | 135 | 13.3 (8.1; 20.3) |
|  | Y5 | 128 | 46.1 (37.2; 55.1) |  | 136 | 42.6 (34.2; 51.4) |  | 130 | 41.5 (33.0; 50.5) |  | 132 | 25.0 (17.9; 33.3) |
| **4** | Post-pri | 169 | 100 (97.8; 100) |  | 174 | 99.4 (96.8; 100) |  | 173 | 98.3 (95.0; 99.6) |  | 170 | 100 (97.9; 100) |
|  | Pre-bst | 153 | 79.1 (71.8; 85.2) |  | 156 | 82.1 (75.1; 87.7) |  | 150 | 80.7 (73.4; 86.7) |  | 153 | 80.4 (73.2; 86.4) |
|  | Post-bst | 158 | 100 (97.7; 100) |  | 153 | 100 (97.6; 100) |  | 160 | 100 (97.7; 100) |  | 152 | 100 (97.6; 100) |
|  | Y2 | 141 | 59.6 (51.0; 67.7) |  | 146 | 56.8 (48.4; 65.0) |  | 144 | 54.9 (46.4; 63.2) |  | 139 | 72.7 (64.5; 79.9) |
|  | Y3 | 136 | 53.7 (44.9; 62.3) |  | 138 | 52.9 (44.2; 61.4) |  | 131 | 49.6 (40.8; 58.5) |  | 136 | 67.6 (59.1; 75.4) |
|  | Y5 | 128 | 39.8 (31.3; 48.9) |  | 135 | 25.2 (18.1; 33.4) |  | 130 | 30.0 (22.3; 38.7) |  | 134 | 53.0 (44.2; 61.7) |
| **5** | Post-pri | 169 | 100 (97.8; 100) |  | 174 | 100 (97.9; 100) |  | 173 | 98.8 (95.9; 99.9) |  | 168 | 2.4 (0.7; 6.0) |
|  | Pre-bst | 149 | 81.9 (74.7; 87.7) |  | 146 | 78.1 (70.5; 84.5) |  | 146 | 78.8 (71.2; 85.1) |  | 148 | 4.7 (1.9; 9.5) |
|  | Post-bst | 157 | 100 (97.7; 100) |  | 153 | 100 (97.6; 100) |  | 160 | 98.8 (95.6; 99.8) |  | 150 | 7.3 (3.7; 12.7) |
|  | Y2 | 140 | 82.1 (74.8; 88.1) |  | 145 | 73.1 (65.1; 80.1) |  | 144 | 76.4 (68.6; 83.1) |  | 140 | 19.3 (13.1; 26.8) |
|  | Y3 | 136 | 77.9 (70.0; 84.6) |  | 138 | 71.0 (62.7; 78.4) |  | 131 | 65.6 (56.9; 73.7) |  | 136 | 22.8 (16.0; 30.8) |
|  | Y5 | 128 | 75.0 (66.6; 82.2) |  | 135 | 65.9 (57.3; 73.9) |  | 130 | 65.4 (56.5; 73.5) |  | 131 | 30.5 (22.8; 39.2) |
| **6B** | Post-pri | 169 | 94.1 (89.4; 97.1) |  | 175 | 88.6 (82.9; 92.9) |  | 173 | 87.3 (81.4; 91.9) |  | 169 | 92.9 (87.9; 96.3) |
|  | Pre-bst | 151 | 82.8 (75.8; 88.4) |  | 155 | 78.1 (70.7; 84.3) |  | 150 | 80.0 (72.7; 86.1) |  | 151 | 58.9 (50.7; 66.9) |
|  | Post-bst | 158 | 98.1 (94.6; 99.6) |  | 153 | 96.7 (92.5; 98.9) |  | 160 | 97.5 (93.7; 99.3) |  | 153 | 99.3 (96.4; 100) |
|  | Y2 | 139 | 71.9 (63.7; 79.2) |  | 146 | 78.8 (71.2; 85.1) |  | 144 | 71.5 (63.4; 78.7) |  | 138 | 91.3 (85.3; 95.4) |
|  | Y3 | 136 | 66.9 (58.3; 74.7) |  | 138 | 68.8 (60.4; 76.4) |  | 133 | 72.2 (63.7; 79.6) |  | 135 | 83.0 (75.5; 88.9) |
|  | Y5 | 128 | 91.4 (85.1; 95.6) |  | 135 | 88.9 (82.3; 93.6) |  | 131 | 87.8 (80.9; 92.9) |  | 131 | 95.4 (90.3; 98.3) |
| **7F** | Post-pri | 169 | 100 (97.8; 100) |  | 175 | 99.4 (96.9; 100) |  | 173 | 98.8 (95.9; 99.9) |  | 169 | 3.0 (1.0; 6.8) |
|  | Pre-bst | 149 | 97.3 (93.3; 99.3) |  | 153 | 92.8 (87.5; 96.4) |  | 148 | 94.6 (89.6; 97.6) |  | 151 | 2.0 (0.4; 5.7) |
|  | Post-bst | 158 | 100 (97.7; 100) |  | 152 | 100 (97.6; 100) |  | 160 | 100 (97.7; 100) |  | 151 | 1.3 (0.2; 4.7) |
|  | Y2 | 141 | 92.2 (86.5; 96.0) |  | 146 | 88.4 (82.0; 93.1) |  | 144 | 85.4 (78.6; 90.7) |  | 140 | 18.6 (12.5; 26.0) |
|  | Y3 | 136 | 80.9 (73.3; 87.1) |  | 138 | 77.5 (69.7; 84.2) |  | 131 | 76.3 (68.1; 83.3) |  | 136 | 24.3 (17.3; 32.4) |
|  | Y5 | 127 | 79.5 (71.5; 86.2) |  | 137 | 79.6 (71.8; 86.0) |  | 130 | 77.7 (69.6; 84.5) |  | 132 | 43.9 (35.3; 52.8) |
| **9V** | Post-pri | 169 | 98.8 (95.8; 99.9) |  | 175 | 97.7 (94.3; 99.4) |  | 173 | 98.3 (95.0; 99.6) |  | 169 | 98.8 (95.8; 99.9) |
|  | Pre-bst | 149 | 95.3 (90.6; 98.1) |  | 155 | 94.8 (90.1; 97.7) |  | 149 | 89.3 (83.1; 93.7) |  | 152 | 94.7 (89.9; 97.7) |
|  | Post-bst | 158 | 99.4 (96.5; 100) |  | 153 | 99.3 (96.4; 100) |  | 160 | 100 (97.7; 100) |  | 153 | 100 (97.6; 100) |
|  | Y2 | 141 | 87.9 (81.4; 92.8) |  | 146 | 82.2 (75.0; 88.0) |  | 144 | 80.6 (73.1; 86.7) |  | 140 | 88.6 (82.1; 93.3) |
|  | Y3 | 136 | 77.9 (70.0; 84.6) |  | 137 | 74.5 (66.3; 81.5) |  | 131 | 70.2 (61.6; 77.9) |  | 136 | 84.6 (77.4; 90.2) |
|  | Y5 | 128 | 73.4 (64.9; 80.9) |  | 137 | 72.3 (64.0; 79.6) |  | 131 | 64.1 (55.3; 72.3) |  | 132 | 77.3 (69.2; 84.1) |
| **14** | Post-pri | 169 | 100 (97.8; 100) |  | 175 | 100 (97.9; 100) |  | 173 | 100 (97.9; 100) |  | 169 | 99.4 (96.7; 100) |
|  | Pre-bst | 153 | 92.2 (86.7; 95.9) |  | 158 | 94.9 (90.3; 97.8) |  | 152 | 90.8 (85.0; 94.9) |  | 153 | 96.1 (91.7; 98.5) |
|  | Post-bst | 158 | 99.4 (96.5; 100) |  | 153 | 100 (97.6; 100) |  | 160 | 99.4 (96.6; 100) |  | 153 | 100 (97.6; 100) |
|  | Y2 | 141 | 91.5 (85.6; 95.5) |  | 146 | 95.9 (91.3; 98.5) |  | 144 | 91.7 (85.9; 95.6) |  | 140 | 98.6 (94.9; 99.8) |
|  | Y3 | 136 | 94.9 (89.7; 97.9) |  | 138 | 97.1 (92.7; 99.2) |  | 132 | 93.2 (87.5; 96.8) |  | 136 | 99.3 (96.0; 100) |
|  | Y5 | 128 | 100 (97.2; 100) |  | 137 | 98.5 (94.8; 99.8) |  | 130 | 96.2 (91.3; 98.7) |  | 133 | 100 (97.3; 100) |
| **18C** | Post-pri | 169 | 98.8 (95.8; 99.9) |  | 175 | 98.9 (95.9; 99.9) |  | 173 | 99.4 (96.8; 100) |  | 169 | 98.8 (95.8; 99.9) |
|  | Pre-bst | 151 | 87.4 (81.0; 92.3) |  | 153 | 93.5 (88.3; 96.8) |  | 145 | 83.4 (76.4; 89.1) |  | 151 | 84.1 (77.3; 89.5) |
|  | Post-bst | 157 | 100 (97.7; 100) |  | 152 | 100 (97.6; 100) |  | 160 | 100 (97.7; 100) |  | 152 | 100 (97.6; 100) |
|  | Y2 | 141 | 84.4 (77.3; 90.0) |  | 146 | 96.6 (92.2; 98.9) |  | 143 | 84.6 (77.6; 90.1) |  | 139 | 86.3 (79.5; 91.6) |
|  | Y3 | 136 | 80.9 (73.3; 87.1) |  | 138 | 93.5 (88.0; 97.0) |  | 132 | 79.5 (71.7; 86.1) |  | 136 | 77.9 (70.0; 84.6) |
|  | Y5 | 128 | 73.4 (64.9; 80.9) |  | 137 | 86.9 (80.0; 92.0) |  | 130 | 72.3 (63.8; 79.8) |  | 132 | 72.7 (64.3; 80.1) |
| **19F** | Post-pri | 169 | 98.2 (94.9; 99.6) |  | 175 | 99.4 (96.9; 100) |  | 173 | 98.8 (95.9; 99.9) |  | 170 | 100 (97.9; 100) |
|  | Pre-bst | 143 | 95.8 (91.1; 98.4) |  | 146 | 95.9 (91.3; 98.5) |  | 139 | 87.8 (81.1; 92.7) |  | 150 | 48.0 (39.8; 56.3) |
|  | Post-bst | 158 | 99.4 (96.5; 100) |  | 151 | 99.3 (96.4; 100) |  | 160 | 100 (97.7; 100) |  | 152 | 98.7 (95.3; 99.8) |
|  | Y2 | 141 | 98.6 (95.0; 99.8) |  | 146 | 95.2 (90.4; 98.1) |  | 144 | 93.1 (87.6; 96.6) |  | 139 | 82.7 (75.4; 88.6) |
|  | Y3 | 136 | 90.4 (84.2; 94.8) |  | 138 | 88.4 (81.9; 93.2) |  | 133 | 91.7 (85.7; 95.8) |  | 136 | 88.2 (81.6; 93.1) |
|  | Y5 | 128 | 96.1 (91.1; 98.7) |  | 137 | 96.4 (91.7; 98.8) |  | 130 | 96.9 (92.3; 99.2) |  | 133 | 96.2 (91.4; 98.8) |
| **23F** | Post-pri | 169 | 95.9 (91.7; 98.3) |  | 175 | 96.0 (91.9; 98.4) |  | 173 | 92.5 (87.5; 95.9) |  | 169 | 94.1 (89.4; 97.1) |
|  | Pre-bst | 152 | 85.5 (78.9; 90.7) |  | 156 | 82.7 (75.8; 88.3) |  | 150 | 75.3 (67.6; 82.0) |  | 153 | 77.1 (69.6; 83.5) |
|  | Post-bst | 158 | 98.7 (95.5; 99.8) |  | 153 | 99.3 (96.4; 100) |  | 160 | 98.8 (95.6; 99.8) |  | 153 | 99.3 (96.4; 100) |
|  | Y2 | 140 | 79.3 (71.6; 85.7) |  | 146 | 80.8 (73.5; 86.9) |  | 144 | 75.7 (67.9; 82.4) |  | 140 | 91.4 (85.5; 95.5) |
|  | Y3 | 136 | 75.0 (66.9; 82.0) |  | 138 | 81.2 (73.6; 87.3) |  | 133 | 73.7 (65.3; 80.9) |  | 136 | 87.5 (80.7; 92.5) |
|  | Y5 | 128 | 86.7 (79.6; 92.1) |  | 136 | 88.2 (81.6; 93.1) |  | 130 | 87.7 (80.8; 92.8) |  | 133 | 94.7 (89.5; 97.9) |

ATP=according-to-protocol; N= number of children with available results; LL=lower limit of the 95% confidence interval; UL=upper limit of the 95% confidence interval; post-pri=1 month after the 3rd dose of primary vaccination; pre-bst=before the booster dose; post-bst=1 month after the booster dose; Y=number of years following booster vaccination.

**Table S5. Percentages of children with serotype-specific pneumococcal OPA titers ≥ 8 by timepoint (ATP cohorts for the respective timepoints) (Study B)**

| Sero-type | Timepoint | **PHiD-CV/MenC-CRM** | |  | **PHiD-CV/MenC-TT** | |  | **PHiD-CV/HibMenC-TT** | |  | **7vCRM/HibMenC-TT** | |
| --- | --- | --- | --- | --- | --- | --- | --- | --- | --- | --- | --- | --- |
|  |  | N | % (LL; UL) |  | N | % (LL; UL) |  | N | % (LL; UL) |  | N | % (LL; UL) |
| **1** | Post-pri | 162 | 54.3 (46.3; 62.2) |  | 168 | 51.2 (43.4; 59.0) |  | 161 | 50.3 (42.3; 58.3) |  | 156 | 1.3 (0.2; 4.6) |
|  | Pre-bst | 144 | 31.9 (24.4; 40.2) |  | 145 | 26.9 (19.9; 34.9) |  | 141 | 29.1 (21.7; 37.3) |  | 143 | 5.6 (2.4; 10.7) |
|  | Post-bst | 140 | 94.3 (89.1; 97.5) |  | 139 | 95.7 (90.8; 98.4) |  | 140 | 90.7 (84.6; 95.0) |  | 137 | 8.8 (4.6; 14.8) |
|  | Y2 | 131 | 22.9 (16.0; 31.1) |  | 131 | 24.4 (17.3; 32.7) |  | 131 | 22.1 (15.4; 30.2) |  | 131 | 10.7 (6.0; 17.3) |
|  | Y3 | 131 | 25.2 (18.0; 33.5) |  | 134 | 23.9 (16.9; 32.0) |  | 123 | 16.3 (10.2; 24.0) |  | 128 | 9.4 (4.9; 15.8) |
| **4** | Post-pri | 154 | 100 (97.6; 100) |  | 159 | 100 (97.7; 100) |  | 159 | 97.5 (93.7; 99.3) |  | 154 | 100 (97.6; 100) |
|  | Pre-bst | 104 | 58.7 (48.6; 68.2) |  | 121 | 55.4 (46.1; 64.4) |  | 112 | 54.5 (44.8; 63.9) |  | 109 | 67.0 (57.3; 75.7) |
|  | Post-bst | 137 | 100 (97.3; 100) |  | 134 | 100 (97.3; 100) |  | 140 | 100 (97.4; 100) |  | 135 | 100 (97.3; 100) |
|  | Y2 | 126 | 42.9 (34.1; 52.0) |  | 127 | 37.8 (29.3; 46.8) |  | 129 | 33.3 (25.3; 42.2) |  | 128 | 60.2 (51.1; 68.7) |
|  | Y3 | 127 | 38.6 (30.1; 47.6) |  | 132 | 37.9 (29.6; 46.7) |  | 118 | 30.5 (22.4; 39.7) |  | 126 | 57.1 (48.0; 65.9) |
| **5** | Post-pri | 153 | 92.8 (87.5; 96.4) |  | 163 | 86.5 (80.3; 91.3) |  | 159 | 88.7 (82.7; 93.2) |  | 153 | 2.0 (0.4; 5.6) |
|  | Pre-bst | 116 | 66.4 (57.0; 74.9) |  | 114 | 62.3 (52.7; 71.2) |  | 117 | 61.5 (52.1; 70.4) |  | 124 | 2.4 (0.5; 6.9) |
|  | Post-bst | 124 | 97.6 (93.1; 99.5) |  | 130 | 97.7 (93.4; 99.5) |  | 136 | 96.3 (91.6; 98.8) |  | 127 | 3.1 (0.9; 7.9) |
|  | Y2 | 130 | 56.2 (47.2; 64.8) |  | 130 | 36.2 (27.9; 45.0) |  | 129 | 42.6 (34.0; 51.6) |  | 132 | 3.8 (1.2; 8.6) |
|  | Y3 | 129 | 50.4 (41.5; 59.3) |  | 132 | 34.8 (26.8; 43.6) |  | 122 | 32.8 (24.6; 41.9) |  | 128 | 3.9 (1.3; 8.9) |
| **6B** | Post-pri | 148 | 87.8 (81.5; 92.6) |  | 150 | 84.7 (77.9; 90.0) |  | 148 | 81.8 (74.6; 87.6) |  | 151 | 97.4 (93.4; 99.3) |
|  | Pre-bst | 133 | 56.4 (47.5; 65.0) |  | 132 | 45.5 (36.8; 54.3) |  | 133 | 56.4 (47.5; 65.0) |  | 137 | 48.9 (40.3; 57.6) |
|  | Post-bst | 142 | 94.4 (89.2; 97.5) |  | 135 | 94.1 (88.7; 97.4) |  | 142 | 95.1 (90.1; 98.0) |  | 140 | 98.6 (94.9; 99.8) |
|  | Y2 | 128 | 65.6 (56.7; 73.8) |  | 127 | 63.8 (54.8; 72.1) |  | 124 | 62.9 (53.8; 71.4) |  | 131 | 87.8 (80.9; 92.9) |
|  | Y3 | 128 | 72.7 (64.1; 80.2) |  | 129 | 75.2 (66.8; 82.4) |  | 122 | 82.8 (74.9; 89.0) |  | 126 | 88.1 (81.1; 93.2) |
| **7F** | Post-pri | 149 | 100 (97.6; 100) |  | 164 | 98.8 (95.7; 99.9) |  | 158 | 96.8 (92.8; 99.0) |  | 138 | 15.2 (9.7; 22.3) |
|  | Pre-bst | 111 | 98.2 (93.6; 99.8) |  | 122 | 95.9 (90.7; 98.7) |  | 115 | 94.8 (89.0; 98.1) |  | 104 | 40.4 (30.9; 50.5) |
|  | Post-bst | 140 | 100 (97.4; 100) |  | 137 | 100 (97.3; 100) |  | 139 | 100 (97.4; 100) |  | 112 | 47.3 (37.8; 57.0) |
|  | Y2 | 131 | 97.7 (93.5; 99.5) |  | 130 | 100 (97.2; 100) |  | 131 | 100 (97.2; 100) |  | 130 | 96.9 (92.3; 99.2) |
|  | Y3 | 131 | 100 (97.2; 100) |  | 134 | 100 (97.3; 100) |  | 123 | 100 (97.0; 100) |  | 128 | 98.4 (94.5; 99.8) |
| **9V** | Post-pri | 153 | 99.3 (96.4; 100) |  | 153 | 98.7 (95.4; 99.8) |  | 155 | 100 (97.6; 100) |  | 150 | 99.3 (96.3; 100) |
|  | Pre-bst | 133 | 98.5 (94.7; 99.8) |  | 133 | 97.7 (93.5; 99.5) |  | 130 | 93.8 (88.2; 97.3) |  | 130 | 96.9 (92.3; 99.2) |
|  | Post-bst | 143 | 100 (97.5; 100) |  | 139 | 100 (97.4; 100) |  | 143 | 100 (97.5; 100) |  | 137 | 100 (97.3; 100) |
|  | Y2 | 131 | 96.9 (92.4; 99.2) |  | 131 | 100 (97.2; 100) |  | 129 | 98.4 (94.5; 99.8) |  | 132 | 93.9 (88.4; 97.3) |
|  | Y3 | 130 | 96.2 (91.3; 98.7) |  | 132 | 98.5 (94.6; 99.8) |  | 120 | 98.3 (94.1; 99.8) |  | 126 | 96.8 (92.1; 99.1) |
| **14** | Post-pri | 154 | 98.1 (94.4; 99.6) |  | 167 | 97.0 (93.2; 99.0) |  | 160 | 96.3 (92.0; 98.6) |  | 154 | 98.1 (94.4; 99.6) |
|  | Pre-bst | 121 | 95.0 (89.5; 98.2) |  | 125 | 92.8 (86.8; 96.7) |  | 114 | 93.9 (87.8; 97.5) |  | 125 | 99.2 (95.6; 100) |
|  | Post-bst | 137 | 100 (97.3; 100) |  | 138 | 100 (97.4; 100) |  | 138 | 100 (97.4; 100) |  | 134 | 100 (97.3; 100) |
|  | Y2 | 129 | 85.3 (78.0; 90.9) |  | 128 | 92.2 (86.1; 96.2) |  | 128 | 82.8 (75.1; 88.9) |  | 131 | 85.5 (78.3; 91.0) |
|  | Y3 | 126 | 76.2 (67.8; 83.3) |  | 128 | 82.0 (74.3; 88.3) |  | 114 | 83.3 (75.2; 89.7) |  | 121 | 90.9 (84.3; 95.4) |
| **18C** | Post-pri | 155 | 96.8 (92.6; 98.9) |  | 163 | 98.2 (94.7; 99.6) |  | 157 | 91.7 (86.3; 95.5) |  | 149 | 100 (97.6; 100) |
|  | Pre-bst | 126 | 35.7 (27.4; 44.7) |  | 118 | 45.8 (36.6; 55.2) |  | 123 | 27.6 (20.0; 36.4) |  | 123 | 28.5 (20.7; 37.3) |
|  | Post-bst | 121 | 100 (97.0; 100) |  | 129 | 98.4 (94.5; 99.8) |  | 121 | 100 (97.0; 100) |  | 114 | 96.5 (91.3; 99.0) |
|  | Y2 | 126 | 40.5 (31.8; 49.6) |  | 121 | 56.2 (46.9; 65.2) |  | 123 | 35.8 (27.3; 44.9) |  | 122 | 39.3 (30.6; 48.6) |
|  | Y3 | 113 | 30.1 (21.8; 39.4) |  | 118 | 47.5 (38.2; 56.9) |  | 113 | 40.7 (31.6; 50.4) |  | 118 | 38.1 (29.4; 47.5) |
| **19F** | Post-pri | 157 | 98.1 (94.5; 99.6) |  | 165 | 93.9 (89.1; 97.1) |  | 159 | 94.3 (89.5; 97.4) |  | 147 | 90.5 (84.5; 94.7) |
|  | Pre-bst | 127 | 80.3 (72.3; 86.8) |  | 135 | 81.5 (73.9; 87.6) |  | 134 | 70.9 (62.4; 78.4) |  | 134 | 21.6 (15.0; 29.6) |
|  | Post-bst | 137 | 97.1 (92.7; 99.2) |  | 137 | 96.4 (91.7; 98.8) |  | 139 | 100 (97.4; 100) |  | 133 | 98.5 (94.7; 99.8) |
|  | Y2 | 130 | 80.8 (72.9; 87.2) |  | 130 | 83.1 (75.5; 89.1) |  | 131 | 67.9 (59.2; 75.8) |  | 132 | 68.9 (60.3; 76.7) |
|  | Y3 | 131 | 66.4 (57.6; 74.4) |  | 133 | 66.2 (57.5; 74.1) |  | 123 | 65.0 (55.9; 73.4) |  | 128 | 69.5 (60.8; 77.4) |
| **23F** | Post-pri | 146 | 95.2 (90.4; 98.1) |  | 156 | 94.9 (90.1; 97.8) |  | 146 | 90.4 (84.4; 94.7) |  | 148 | 99.3 (96.3; 100) |
|  | Pre-bst | 132 | 91.7 (85.6; 95.8) |  | 136 | 88.2 (81.6; 93.1) |  | 128 | 82.0 (74.3; 88.3) |  | 137 | 90.5 (84.3; 94.9) |
|  | Post-bst | 145 | 100 (97.5; 100) |  | 143 | 100 (97.5; 100) |  | 146 | 100 (97.5; 100) |  | 143 | 100 (97.5; 100) |
|  | Y2 | 125 | 84.8 (77.3; 90.6) |  | 124 | 90.3 (83.7; 94.9) |  | 125 | 90.4 (83.8; 94.9) |  | 132 | 97.7 (93.5; 99.5) |
|  | Y3 | 129 | 76.0 (67.7; 83.1) |  | 133 | 81.2 (73.5; 87.5) |  | 119 | 72.3 (63.3; 80.1) |  | 121 | 91.7 (85.3; 96.0) |

**Note:** Testing of *S. pneumoniae* opsonophagocytic activity (OPA) was not performed at year 5. ATP=according-to-protocol; N=number of children with available results; LL=lower limit of the 95% confidence interval; UL=upper limit of the 95% confidence interval; post-pri=1 month after the 3rd dose of primary vaccination; pre-bst=before the booster dose; post-bst=1 month after the booster dose; Y=number of years following booster vaccination.
